# Do sugar-sweetened beverages cause adverse health outcomes in children? A systematic review protocol

**DOI:** 10.1186/2046-4053-3-96

**Published:** 2014-09-04

**Authors:** Adrienne Stevens, Candyce Hamel, Kavita Singh, Mohammed T Ansari, Esther Myers, Paula Ziegler, Brian Hutton, Arya Sharma, Lise M Bjerre, Shannon Fenton, Robert Gow, Stasia Hadjiyannakis, Kathryn O’Hara, Catherine Pound, Erinn Salewski, Ian Shrier, Noreen Willows, David Moher, Mark Tremblay

**Affiliations:** 1 Clinical Epidemiology Program, Centre for Practice-Changing Research, Ottawa Hospital Research Institute, 501 Smyth Road, Box 201B, Ottawa, ON K1H 8L6, Canada; 2 University of Ottawa, 30 Marie Curie Street, Ottawa K1N 6N5, Canada; 3 E F Myers Consulting Inc, 600 North Oak Street, Trenton, IL 62293, USA; 4 Research Evidence Analysis, Academy of Nutrition and Dietetics, 120 South Riverside Plaza, Suite 2000, Chicago, IL 60606, USA; 5 Canadian Obesity Network, Royal Alexandra Hospital, MMC, Room 102, 10240 Kingsway Avenue, Edmonton, AB T5H 3V9, Canada; 6 C.T. Lamont Primary Health Care Research Centre, Bruyere Research Institute, Department of Family Medicine, University of Ottawa, 43 Bruyere Street, Annex E, Room 206, Ottawa, ON K1N 5C8, Canada; 7 Planning, Research and Analysis Branch, Ministry of Health and Long-Term Care, 80 Grosvenor Street, 8th Floor, Hepburn Block, Toronto, ON M7A 1R3, Canada; 8 Division of Cardiology, Children’s Hospital of Eastern Ontario, 401 Smyth Road, Ottawa, ON K1H 8L1, Canada; 9 Division of Endocrinology, Department of Pediatrics and Department of Medicine, University of Ottawa, 401 Smyth Road, Ottawa, ON K1H 8L1, Canada; 10 School of Journalism and Communication Journalism, Carleton University, 4th Floor River Building, 1125 Colonel By Drive, Ottawa, ON K1S 5B6, Canada; 11 Division of Pediatric Medicine, Children's Hospital of Eastern Ontario and Department of Medicine, University of Ottawa, 401 Smyth Road, Ottawa, ON K1H 8L1, Canada; 12 Healthy Living Section, Chronic Disease and Injury Prevention, Ottawa Public Health, 100 Constellation Drive, 7th Floor East (26-42), Ottawa, ON K2G 6J8, Canada; 13 Centre for Clinical Epidemiology, Lady Davis Institute for Medical Research, Jewish General Hospital, McGill University, 3755 Cote Sainte-Catherine Road, Montreal, QC H3T 1E2, Canada; 14 Department of Agricultural, Food and Nutritional Science, University of Alberta, 4-378 Edmonton Clinic Health Academy, Mailbox #54, 11405 87 Avenue, Edmonton, AB T6G 2P5, Canada; 15 Department of Epidemiology and Community Medicine, University of Ottawa, 501 Smyth Road, Box 201B, Ottawa, ON K1H 8L6, Canada; 16 Healthy Active Living and Obesity Research Group, Children’s Hospital of Eastern Ontario Research Institute, 401 Smyth Road, Ottawa, ON K1H 8L1, Canada

**Keywords:** Sugar-sweetened beverage, Paediatric, Child, Chronic disease, Academic achievement, Dental caries, Obesity, Type 2 diabetes, Systematic review

## Abstract

**Background:**

Cardiovascular disease and type 2 diabetes are examples of chronic diseases that impose significant morbidity and mortality in the general population worldwide. Most chronic diseases are associated with underlying preventable risk factors, such as elevated blood pressure, high blood glucose or glucose intolerance, high lipid levels, physical inactivity, excessive sedentary behaviours, and overweight/obesity. The occurrence of intermediate outcomes during childhood increases the risk of disease in adulthood. Sugar-sweetened beverages are known to be significant sources of additional caloric intake, and given recent attention to their contribution in the development of chronic diseases, a systematic review is warranted. We will assess whether the consumption of sugar-sweetened beverages in children is associated with adverse health outcomes and what the potential moderating factors are.

**Methods/Design:**

Of interest are studies addressing sugar-sweetened beverage consumption, taking a broad perspective. Both direct consumption studies as well as those evaluating interventions that influence consumption (e.g. school policy, educational) will be relevant. Non-specific or multi-faceted behavioural, educational, or policy interventions may also be included subject to the level of evidence that exists for the other interventions/exposures. Comparisons of interest and endpoints of interest are pre-specified. We will include randomized controlled trials, controlled clinical trials, interrupted time series studies, controlled before-after studies, prospective and retrospective comparative cohort studies, case–control studies, and nested case–control designs. The MEDLINE®, Embase, The Cochrane Library, CINAHL, ERIC, and PsycINFO® databases and grey literature sources will be searched. The processes for selecting studies, abstracting data, and resolving conflicts are described. We will assess risk of bias using design-specific tools. To determine sets of confounding variables that should be adjusted for, we have developed causal directed acyclic graphs and will use those to inform our risk of bias assessments. Meta-analysis will be conducted where appropriate; parameters for exploring statistical heterogeneity and effect modifiers are pre-specified. The Grading of Recommendations Assessment, Development and Evaluation (GRADE) approach will be used to determine the quality of evidence for outcomes.

**Systematic review registration:**

PROSPERO CRD42014009641.

## Background

Cardiovascular disease and type 2 diabetes are examples of chronic diseases that cause significant morbidity and mortality in the general population worldwide. Effective interventions and public health policies are thus required to address the burden of chronic disease. Most chronic diseases are associated with underlying preventable risk factors, such as elevated blood pressure, high blood glucose or glucose intolerance, hyperlipidemia, physical inactivity, excessive sedentary behaviours, overweight and obesity, and tobacco usage. The development of chronic diseases may be prevented if these risk factors are addressed before they progress to overt disease. A simple, unidirectional schematic depicts the hypothesized pathways by which sugar-sweetened beverage (SSB) consumption leads to the development of overweight/obesity, prediabetes/type 2 diabetes, dyslipidemia, hypertension, dental caries, and fractures and any effects on academic achievement in children (Additional file 1). Overweight/obesity, prediabetes, dyslipidemia, and hypertension are risk factors for the development of cardiovascular/cerebrovascular disease in later adulthood. These mechanisms have not been conclusively established by research studies, and several conflicting theories have been put forward [1-8]. However, as some of the SSB constituents (notably sugar but also caffeine, where added) are postulated to be involved in the mechanisms of disease development, they are included in our depiction of the disease pathways.

The role of SSBs in chronic disease has been a focus in the media in recent years. Well-known is New York City’s proposal to ban the sale of large-sized SSB products greater than 16 oz, including sodas, sweetened teas and coffees, energy drinks, and fruit drinks in restaurants, delis, sports arenas, movie theatres, and food carts [9]. The proposed ban has been twice rejected but is being reviewed by the United States (US) Court of Appeals. Some districts have also banned the sale of soda in schools [9], and the beverage industry has also self-regulated the sales of specific beverages to schools through the School Beverage Guidelines [10]. In March 2014, the World Health Organization (WHO) released draft guidelines with recommendations on limiting sugar consumption (through food and beverage) to reduce public health problems like obesity and dental caries. They are recommending decreasing the total energy intake of sugar by day from 10% (recommended since 2002) to 5% [11].

Some researchers hypothesize that SSBs are significant sources of caloric intake. With the adjusted prevalence of SSB consumption at 66% for children (2–11 years) and 77% among adolescents (12–19 years) according to the 2007–2008 US National Health and Nutrition Examination Survey (NHANES) data, the mean adjusted SSB energy intake was 178 and 286 kcal/day, respectively; intake of sports/energy drinks was 84 and 167 kcal/day, respectively, in these populations [12]. Heavy consumption (≥500 kcal/day) occurred among 5% of children and 16% of adolescent consumers [12]. Soda was the most heavily consumed SSB in adolescents, while fruit drinks were consumed the most in children [12].

Several reviews have presented evidence syntheses on associations between SSB consumption and weight and include studies in children [13-18]. Studies in three reviews have shown a positive effect of SSB intake and weight gain in children [15-17]. Two reviews report mixed results among the subset of studies assessing SSB consumption in children [13,19]. The review by Forshee et al. assessed SSBs and body mass index in children and adolescents and found no association [14]. A World Health Organization report concluded that the evidence to implicate high intake of sugar-sweetened drinks on weight gain is moderately strong and recommends restricting the intake of SSBs in children and adolescents [19]. In addition, in a systematic review conducted as part of the Dietary Guidelines Advisory Committee deliberations, they concluded that ‘strong evidence supports the conclusion that greater intake of sugar-sweetened beverages is associated with increased adiposity in children’ [20].

One review reports a dose–response relationship between SSB and weight status but no corresponding weight loss when SSB consumption was reduced [21]. Assessing studies in adults and children, a systematic review of epidemiological research analysed beverages by category (water, milk, soft drinks, sugary drinks, non-carbonated, fruit juices, carbonated beverages, hot beverages, and alcoholic beverages) and the authors concluded that the results were inconsistent and did not establish an association between beverage intake and subsequent weight gain [22]. Within their beverage categories, however, the authors did not consistently separate SSBs from other drinks; for example, sweetened hot beverages were not differentiated from non-sweetened. Others have reviewed the biological plausibility of SSBs to uniquely affect the physiological energy balance regulatory systems (e.g. satiety and post-prandial regulatory systems) and concluded that known biological mechanisms did not support the concept that SSBs were somehow different from other sources of energy [23].

Other reviews have synthesized evidence in adults and shown effect of SSBs on metabolic syndrome/type 2 diabetes [15,16,24] and cardiovascular disease [15]. It is reasonable to assume that effects on intermediate outcomes, such as insulin resistance, prediabetes, and dyslipidemia may occur in the paediatric population.

The role of SSBs in the context of total caloric intake is unclear. Although total caloric intake and caloric intake from other dietary sources are factors that may be thought of as confounders, some evidence suggests that they may be clustered with SSB consumption and general unhealthy eating habits [15,16,25]. Conflicting evidence exists as to whether SSB intake is associated with increased energy intake [13,20] and may lower the intake of milk, calcium, and other nutrients [13]. Furthermore, given that SSB consumption may alter taste preferences and quality of diet, caloric intake may in fact mediate the effect of SSB consumption and health outcomes (i.e. it may lie in the causal pathway).

The available systematic review evidence is conflicting and presents with several methodological issues, making firm conclusions difficult [26]. The definition of what constitutes an SSB, for example, may vary and may not be explicitly described [13,17,18,27,28]. Reviews may not have accounted for all variables that can confound associations between SSB consumption and health outcomes. Most reviews have addressed singular outcomes of interest to public health. Further, new primary evidence [29-32] on the topic is rapidly accumulating since the last systematic review was published in 2011. To overcome identified methodological challenges, we will carry out a rigorous assessment of the bias of individual studies, which will include an evaluation of confounding and biasing factors using causal directed acyclic graphs (DAGs) [33].

### Objectives

The objective of the systematic review is to answer the following research questions:

In children, does the consumption of SSBs cause adverse health outcomes? If so, what potential moderating factors affect the causal association between SSB consumption and outcomes?

## Methods/Design

### Eligibility criteria

Studies will be selected according to the criteria outlined below.

#### Study designs

We will include randomized controlled trials (RCTs) including cluster RCTs, controlled (non-randomized) clinical trials (CCTs) or cluster trials, interrupted time series (ITS) studies with at least three data points before and after the intervention [34], controlled before-after (CBA) studies, prospective and retrospective comparative cohort studies, and case–control or nested case–control studies. Cluster randomized, cluster non-randomized, or CBA studies will be included only if there are at least two intervention sites and two control sites [34]. All of the included study designs have the potential to provide evidence about causal relationships. We will exclude cross-sectional studies, case series, and case reports.

#### Participants

We will include studies examining the general human paediatric population or healthy human children aged 2–17 years. We will also include studies on children who are overweight or obese, but will otherwise exclude studies of populations restricted to specific diseases, conditions, or metabolic disorders. We will include studies addressing both adults and children if data provided for children are reported separately.

#### Interventions

Of interest are interventions addressing SSB consumption, taking a broad perspective. In addition to direct consumption studies, we would consider interventions that influence consumption, such as those addressing the level of access to SSBs (e.g. school policy) and educational interventions addressing consumption as relevant. Non-specific or multi-faceted behavioural, educational, or policy interventions may also be included subject to the level of evidence that exists for the aforementioned interventions/exposures. We will also consider other types of interventions on a case-by-case basis, subject to what exists in the literature.

In terms of defining an SSB, we view them as akin to a complex intervention because they are composed of several parts. For example, in addition to sugar, some beverages contain caffeine and the by-products of caramel colouring (2-methylimidazole, 4-methylimidazole), which may contribute independently to adverse health outcomes. The scope of the review, therefore, warrants an examination of SSB consumption as a whole, rather than the specific constituents as exposure variables. Otherwise, such evaluations would have necessarily required the inclusion of studies addressing those constituents and in foods and drinks other than SSBs.

We will use the Centers for Disease Control and Prevention (CDC) definition of SSB for drinks that should be included. According to the CDC, SSBs contain added caloric sweeteners [35], which would include natural sweeteners such as honey and concentrated fruit juice. We have developed a classification scheme based on the CDC definition for use during the review (see classification scheme for SSBs below). For beverages such as coffee, tea, and homemade lemonade, studies will be included in the review if they explicitly state that sugar was added. We will exclude artificially sweetened (e.g. with aspartame or sucralose) beverages and 100% fruit or vegetable juices as exposures/interventions.

We will classify SSBs described in studies according to the following broad categories:

• Sodas—caffeinated/non-caffeinated (soft drinks, soda, pop, soda pop).

• Other non-carbonated sweetened beverages (fruitades, fruit drinks, fruit punches, [iced] teas, coffees, non-dairy fruit smoothies)—caffeinated/non-caffeinated.

• Fortified sweetened beverages (energy drinks, fortified waters, sports drinks)—caffeinated/non-caffeinated and containing vitamins, amino acids, herbal stimulants, or other ingredients.

• Flavored/sweetened milk or milk alternative beverages (dairy, soy, almond, milkshakes, dairy-based fruit smoothies)—caffeinated/non-caffeinated.

#### Comparators

Given the broad perspective for interventions of interest, several comparisons will be relevant to include. Some may be more likely to come from observational designs, others from experimental studies.

Direct consumption studies:

1. SSB consumption compared with consumption of non-SSB drink (e.g. 100% fruit juice, artificially sweetened beverage, water)

2. Higher level of SSB consumption versus lower level of SSB consumption for the same drink type (e.g. carbonated cola beverages)

3. Comparisons among different categories of SSBs (e.g. soft drinks compared with fruit drinks; see classification scheme for SSBs) consumed in similar amounts

Interventions that influence consumption:

4. One level of access to SSB compared with another level of access (e.g. school policy on beverages in vending machines)

5. Educational intervention to specifically promote lower or no SSB consumption compared with no educational intervention/regular curriculum coverage/general health-focussed intervention

6. Non-specific or multi-faceted educational, behavioural, or policy dietary intervention (may include component of SSB consumption) compared with no intervention

7. Other comparisons involving interventions that address our research question (interventions assessed on a case-by-case basis, as encountered in the literature)

For comparator groups 2 and 3, we anticipate that volume will be the most feasible to analyse; however, we will extract all measures in which consumption reported (e.g. volume, caloric intake from sugar) in studies to see what analysis is possible.

For feasibility, category 6 comparisons (non-specific, multi-faceted interventions) will be coded at title/abstract screening and not put through to full-text screening. If sparse evidence in the other potential comparison types, we will revisit eligibility for comparison 6.

#### Outcomes

Endpoints important for decision-making are of primary interest. If reported on, these will be analysed and graded. If a given clinical endpoint is not reported on, we will analyse and grade their relevant surrogate outcome.

• Endpoints important for decision-making:

– Academic achievement

– Dyslipidemia

– Fractures

– Hypertension

– Overweight/obesity/change in weight

– Prediabetes/type 2 diabetes

– Dental caries

– Quality of life (generic, validated tools only, such as those in Additional file 2)

• Surrogate outcome:

– Bone mineral density (BMD)

Dental caries will be analysed for all ages, but will be the only outcome for children aged 2–4 years old, as other health effects are unlikely to be seen this early. As some outcomes may be reported as a composite measure, we will extract all composite and individual outcomes as reported in the studies.

Outcomes will be collected as reported, with the exception of quality of life, which will be collected only if assessed with generic (not disease-specific), validated tools. Due to possible variation in disease definitions over time, we will extract definitions of outcomes as reported in individual studies. We will extract outcomes in all data forms (e.g. dichotomous, continuous) as reported in the included studies.

#### Timing

Studies will be selected for inclusion based on the length of follow-up of outcomes. The following will be used as a guide for all study designs:

• For the all decision-making endpoint outcomes, studies should have a follow-up time of at least 1 year.

• For the surrogate outcome BMD, studies should be at least 6 months duration for follow-up.

#### Setting

There will be no restrictions by type of setting.

#### Language

We will include articles reported in the English and French languages. A list of possibly relevant titles in other languages will be provided as an appendix.

### Search methods

#### Electronic searches

A comprehensive literature search using high-recall subject searches will be conducted in MEDLINE®, Embase, CINAHL, PsycINFO®, ERIC, and The Cochrane Library. Electronic search strategies (Additional file 3) have been developed by an experienced information specialist and peer-reviewed according to the peer review of electronic search strategies (PRESS) guidelines [36]. The search will not be restricted for time period or the language of publication. We will exclude comments, letters, and editorials.

#### Other sources

Grey literature sources, such as websites listed within the Canadian Agency for Drugs and Technologies in Health’s (CADTH) Grey Matters checklist, the Federation of American Societies for Experimental Biology (FASEB) meeting abstracts, the Obesity Society abstracts, the Food and Nutrition Conference and Expo (FNCE) abstracts, the International Diabetes Federation website, the Centers for Disease Control and Prevention website, the Rudd Center for Food Policy and Obesity website, the Obesity and Energetics Offerings website, the American Heart Association, the European Union, the Center for Science in the Public Interest, the 20th International Congress of Nutrition, the American Beverage Association, and Refreshments Canada will be searched. We will consult within the research team for relevant studies that may not be in the published literature. We will scan the references of included studies and relevant reviews for additional articles and perform a forward search on key articles. We will search ClinialTrials.gov, the WHO International Clinical Trials Registry Platform, and the International Association for the Study of Obesity (IASO) for completed and ongoing studies.

#### Study selection process

Literature search results will be de-duplicated in Reference Manager [37] before uploading to Distiller Systematic Review Software® (Distiller SR), an online program that facilitates screening and data extraction [38]. Screening questions will be developed and pilot-tested with a subset of records before implementation. All titles and abstracts of records will be screened by one person; those deemed not relevant will be verified by a second person for exclusion. Full-text reports for all potentially relevant records and those without an available abstract will be screened by two independent reviewers. Discrepancies will be resolved by consensus or a third person. The study selection process will be reported using the Preferred Reporting Items for Systematic Reviews and Meta-Analyses (PRISMA) flow diagram [39], including reasons for excluding full-text articles.

### Data collection and analysis

#### Data extraction and management

Feedback will be solicited from the research team on the draft list of data variables for extraction. Data extraction forms will be developed and pilot-tested in Distiller SR. One person will extract all information. A second person will verify 20% of studies for general characteristics information and 100% of studies regarding outcomes data. Disagreements will be resolved by consensus or by a third team member, if needed. Information on the descriptive and quantitative characteristics of studies will include the following:

• Publication details (e.g. year of publication, language, publication status).

• Characteristics of study (e.g. study design, methods, country, setting, sample size, number of centres [if applicable], duration of follow-up, source of funding).

• Characteristics of population (e.g. age, gender, ethnicity, co-interventions, information regarding respondent bias/representativeness of the included population).

• Details about the exposure/intervention (e.g. type and category of SSB [see classification scheme for SSBs]), brand name, amount of sugar/serving, type of added sugar, amount of caffeine, other specific ingredients and their quantification, frequency of use, amount consumed in millilitres, SSB caloric content, percent of total calories obtained from SSB consumption, method of assessing SSB consumption; type of educational or other interventions and description, type of professional delivering intervention)

• Details about comparator group (e.g. for beverages: identity, frequency of use, amount used, brand name, specific ingredients)

• Outcomes of interest for the longest duration of follow-up (definitions, measurement methods, data, adjusted and unadjusted effect estimates)

• Confounding factors that were taken into consideration

• Risk of bias items

#### Assessing the risk of bias

The risk of bias for each included study will be assessed by one member of the research team and verified by a second member. Disagreements will be resolved by consensus or by a third team member, if needed. Assessment tool questions were reorganized (as needed) to ensure that domains relating to selection, confounding (where applicable), performance, attrition, detection, reporting, and ‘other’ biases were addressed in all tools. A modified version of the Cochrane Risk of Bias tool will be used to evaluate RCTs (Additional file 4) [40]. In addition to the standard domains of bias, we will assess cluster trials for the possibility of recruitment bias [41]. A modified version of the Academy of Nutrition and Dietetics’ Evidence Analysis Library (EAL) Quality Criteria Checklist will be used to evaluate observational studies and CCTs (Additional file 4) [42]. We removed questions pertaining solely to reporting characteristics, and we added a few other relevant questions. To evaluate ITS and CBA studies, a modified Cochrane Effective Practice and Organisation of Care (EPOC) tool will be used (Additional file 4) [43]. Study sponsorship will be assessed for all studies. Each domain within a tool will be judged as unclear, low, or high risk of bias, with supporting information provided from the report or reviewer interpretation to rationalize the judgement of bias [40]. Some domains are outcome-specific and will be assessed at the outcome level. The risk of bias for outcomes will be factored into grading the quality of evidence. The overall risk of bias for the body of evidence will involve a judgement of the relative importance of domains, guided by known empirical evidence of bias, the likely direction of bias, and the likely magnitude of bias [40]. We will follow the Grading of Recommendations Assessment, Development and Evaluation (GRADE) guidance for determining the extent of the risk of bias for the body of evidence [44].

Step 1 - Assessment of risk of bias for individual studies for a given outcome:

• Low risk of bias: when all key domains are at a low risk of bias.

• Moderate risk of bias: crucial limitation for one domain or some limitations for multiple domains sufficient to lower confidence in the effect estimate.

• High risk of bias: crucial limitation for one or more domains sufficient to substantially lower confidence in the effect estimate.

Step 2 - Assessment of risk of bias for the body of evidence across studies for a given outcome (incorporated into GRADE assessments as one of the required domains):

• No serious limitation, do not downgrade: body of evidence mostly from studies at a low risk of bias.

• Serious limitations, downgrade one level: body of evidence mostly from studies at moderate risk of bias.

• Very serious limitations, downgrade two levels: body of evidence mostly from studies at high risk of bias.

Regarding confounding bias, causal DAGs will be used [33]. Causal DAGs are graphical models used in epidemiology to determine sets of confounders that should be adjusted for to obtain unbiased effect estimates. They also identify biasing paths associated with selection bias. By adjusting for only those confounders that are derived from a causal DAG, the potential for over-adjustment or the creation of selection bias by conditioning on colliders (i.e. variables that are common effects of both the exposure and outcome) are reduced, given that the causal DAG is correct [45,46]. We will use the minimal sufficient adjustment sets generated from the causal DAGs as a guide to determine if studies have adequately accounted for confounding variables or created selection bias by over-adjustment of imbalances between exposure and control groups. For a given outcome, if studies differentially account for variables from the minimum adjustment sets, we may not pool those studies in a meta-analysis. Draft causal DAGs (Additional file 5) have been developed using the DAGitty program [47]. The causal DAGs may be modified to include important and justifiable variables we encounter when reviewing included studies.

#### Dealing with missing data

If information or data are missing or incomplete, we will attempt to contact the study authors twice over 2 weeks by email. If feasible, we will incorporate loss-to-follow-up data. We will not impute effect estimates, but will impute missing standard deviations or standard errors using data from other similar studies in the review, using an approach suggested in the literature [48].

### Data analyses

For dichotomous outcomes, the risk ratio or odds ratio and 95% confidence intervals will be used for pooling. For continuous outcomes, mean differences and 95% confidence intervals will be used for pooling for outcomes reported on the same scales or measured in the same units. Standardized mean differences will be used for pooling where continuous outcomes are reported using different scales or measures. Transformation of data to allow analyses with mean differences will be made, wherever possible. All formats of continuous outcome data will be extracted whether reported as post-intervention or change from baseline. We will consider using the *r* value, a correlation coefficient, for continuous variables when some studies analyse as a dichotomous outcome and others analyse as a continuous outcome. Statistics from individual studies will be converted to an *r* value before meta-analysing [49,50]. The *r* value can be roughly interpreted as a small (*r* = 0.1), medium (*r* = 0.3), or large (*r* = 0.5) effect size. We plan to transform the pooled *r* to another statistic, such as an odds ratio, to aid in interpretation [49]. For time-to-event data, the hazard ratio, which is usually estimated from a Cox proportional hazards model, will be pooled using the generic inverse variance method [51]. Comprehensive Meta-Analysis will be used for meta-analyses [52]. SAS software will be used for meta-regression analyses [53].

#### Other statistical considerations

##### Sparse binary data and studies with zero events

When studies report rare events, narrative synthesis will suffice. When event rates are less than 1%, the Peto odds ratio method will be used. However, when control groups are of unequal sizes, when large magnitude of effect is observed, or when events become more frequent (5%–10%), the Mantel-Haenszel method without correction factor will be employed for quantitative synthesis [51].

##### Data conversions

Where needed, we will convert data (e.g. standard error to standard deviation) for use in analyses and to facilitate consistent presentation of results across studies.

##### Interrupted time series designs

If interrupted time series studies are included, we will re-analyse data, where needed and if feasible, for change in level and slope according to time series regression analyses [54].

### Evidence synthesis

Study characteristics will be summarized narratively in the text and shown in summary tables in the report. Before meta-analyses are performed, studies will be assessed for heterogeneity on clinical and methodological characteristics; we plan to review these decisions with the research team before conducting analyses. For outcomes that can be measured on various scales (e.g. quality of life), heterogeneity of outcome measurements will also be assessed before pooling. With sufficient homogeneity and quantity of data, we will pool studies using standard random effects meta-analytic methods [55,56]. Any meta-analyses will be done separately for observational studies and experimental ones. Narrative synthesis of data will be conducted when quantitative pooling is considered inappropriate (team decision based on the aforementioned issues, such as disparate clinical characteristics of included patients/participants). When important clinical or methodological heterogeneity precludes pooling, we may still present forest plots without a pooled summary estimate to show individual study effects. Effect estimates from observational studies at a high risk of bias may be excluded from the evidence synthesis when their findings are inconsistent with studies at a lower risk of bias. If only observational studies at a high risk of bias exist for a given outcome, we will not synthesize those studies because they are unlikely to inform about causality.

#### Statistical heterogeneity and effect modifiers

Statistical heterogeneity will be assessed using Cochrane *Q* (considered statistically significant at *p* < 0.10) and *I*-squared statistics. For the interpretation of *I*-squared, a rough guide of low (0%–25%), moderate (25%–50%), substantial (50%–75%), and considerable (75%–100%) heterogeneity will be used [57,58]. When a body of evidence is determined to be statistically heterogeneous, we plan to explore the impact of moderating factors using a combination of subgroup analysis and meta-regression techniques, where the optimal approach for each variable will be determined once we see how data are reported in studies. Some variables (*) will be investigated as potential effect modifiers regardless of heterogeneity tests. The distribution of several aspects of patient demographics, exposure, and other characteristics will be of interest and include the following:

• Participant age*

• Sex*

• Tanner stage*

• Ethnicity*

• Region

• Overweight/obesity*

• SSB category*

• Amount of SSB consumption*

• Type of comparator*

• Caffeine-containing drinks*

• Outcome definition

• Study design

• Duration of study

• Single versus multi-centre studies

• Risk of bias assessments

• Covariate adjustment

• Funding

• Adjustment for total caloric intake*.

We will follow previously published guidance for meta-regression [51]. Meta-regression will be based on random effects model to allow for residual unexplained heterogeneity. A *p* value <0.10 will characterize statistical significance. When the sizes of the included studies are moderate or large, there should be at least 10 studies for a continuous study-level variable. For a categorical subgroup variable, each subgroup should have a minimum of four studies. These numbers serve as the lower bounds for considering meta-regression [51]. When included studies are mostly small in size, univariable meta-regression will be used when an insufficient number of studies are available to conduct multivariable analyses.

Regarding caloric intake, if highly correlated with SSB consumption, it will be difficult to decipher the independent effect of SSB consumption. If caloric intake lies in the causal pathway, then adjusting for it would eliminate any true associations between SSB consumption and outcomes. However, a counter-argument could be made that failure to adjust for caloric intake would produce spurious positive associations. Due to this uncertainty, we reasoned that assessing effect estimates for this variable in a subgroup analysis was the most appropriate; studies will not be penalized for adjusting or not adjusting in assessments of confounding bias.

#### Sensitivity analysis

Sensitivity analyses may be used to restrict analyses to low risk of bias and any decisions made regarding data handling.

#### Assessing for small study effects

We will investigate small study effects by the performance of cumulative meta-analysis (studies ordered and synthesized from the most to the least precise) and/or other graphical or statistical techniques if the following criteria are met: there are at least 10 studies available, studies are of unequal sizes, there are no substantial clinical and methodological differences between smaller and larger studies, and quantitative results are accompanied with measures of dispersion [59-62].

## Discussion

### Grading the quality of evidence and interpretation

We will use the GRADE approach to evaluate the quality of evidence for outcomes [44]. Quality of evidence is the level of confidence for a causal inference that authors place in the estimate of effect for an outcome (i.e. their judgement that the evidence reflects the true effect). As stated previously, if surrogate measures are analysed in lieu of decision-making endpoints, these will be graded but downgraded for indirectness.

When grading the evidence, reviewers will evaluate the domains of study limitations (risk of bias), inconsistency, indirectness, imprecision, and publication bias and downgrade where important limitations exist. Studies may also be upgraded based on a strong magnitude of effect that is not due to known biases, dose–response gradient, and residual confounding that would have reduced the observed effect. The overall quality of evidence grade will be designated as high (confident the true effect lies close to that of the estimate), moderate (moderately confident in the effect estimate but may be substantially different), low (confidence in the effect estimate is limited), or very low (very little confidence in the effect estimate) [44]. The results will be discussed in light of the strength of findings as well as their implications for research and public health.

### Quality assurance

We used the PRISMA for Protocols (PRISMA-P) checklist for reporting this protocol [63]. This review will be reported according to the PRISMA statement [39] and using A Measurement Tool to Assess the Methodological Quality of Systematic Reviews (AMSTAR) tool for additional quality control [64]. This protocol does not update any previously conducted systematic review. Any amendments made to this protocol when conducting the review will be outlined in the review’s manuscript.

## Abbreviations

AMSTAR: A Measurement Tool to Assess the Methodological Quality of Systematic Reviews; BMD: bone mineral density; CADTH: Canadian Agency for Drugs and Technologies in Health; CBA: controlled before-after; CCTs: controlled clinical trials; CDC: Centers for Disease Control and Prevention; CINAHL: Cumulative Index to Nursing and Allied Health Literature; DAGs: directed acyclic graphs; Distiller SR: Distiller Systematic Review Software; EAL: Evidence Analysis Library; EPOC: Effective Practice and Organisation of Care; ERIC: Education Resources Information Center; FASEB: Federation of American Societies for Experimental Biology; FNCE: Food and Nutrition Conference and Expo; GRADE: Grading of Recommendations Assessment, Development and Evaluation; IASO: International Association for the Study of Obesity; ITS: interrupted time series; NHANES: National Health and Nutrition Examination Survey; PRESS: peer review of electronic search strategies; PRISMA: Preferred Reporting Items for Systematic Reviews and Meta-Analyses; PRISMA-P: Preferred Reporting Items for Systematic Reviews and Meta-Analysis for Protocols; RCTs: randomized controlled trials; SSB: sugar-sweetened beverage; WHO: World Health Organization.

## Competing interests

The authors declare that they have no competing interests.

## Authors’ contributions

AdS, CH, KS, MTA, EM, PZ, BH, DM, and MT drafted the protocol. AS, LMB, SF, RG, SH, KOH, CP, ES, IS, and NW reviewed the draft critically and provided important feedback on the content. MT is the guarantor. All authors approved the final manuscript.

## Supplementary Material

Additional file 1 **Postulated physiologic mechanisms regarding disease development due to SSB consumption **[65]**-**[72]**.** This file provides a description of the postulated physiologic mechanisms of disease development from the consumption of sugar-sweetened beverages. Click here for file

Additional file 2 **Examples of validated generic quality of life instruments.** This file provides examples of validated generic quality of life instruments. Click here for file

Additional file 3 **Search strategy for the Embase, MEDLINE, and PsycINFO databases.** This file provides the search strategies used for the various bibliographic databases. Click here for file

Additional file 4 **Risk of Bias Assessment.** This file provides the tools and domains for risk of bias assessment. Click here for file

Additional file 5 **Causal directed acyclic graphs (DAG) depicting the postulated causal and biasing pathways between sugar-sweetened beverage consumption and adverse health outcomes **[72]**,**[73]**.** This file provides illustrations and accompanying text of the postulated causal and biasing pathways between sugar-sweetened beverage consumption and adverse health outcomes. Click here for file

## References

[B1] Pereira MA The possible role of sugar-sweetened beverages in obesity etiology: a review of the evidence Int J Obes (Lond) 2006 30 Suppl 3 S28 S36

[B2] Basciano H Federico L Adeli K Fructose, insulin resistance, and metabolic dyslipidemia Nutr Metab (Lond) 2005 2 1 5 15723702 10.1186/1743-7075-2-5 PMC552336

[B3] Elliott SS Keim NL Stern JS Teff K Havel PJ Fructose, weight gain, and the insulin resistance syndrome Am J Clin Nutr 2002 76 5 911 922 12399260 10.1093/ajcn/76.5.911

[B4] Hung CT Effects of high-fructose (90%) corn syrup on plasma glucose, insulin, and C-peptide in non-insulin-dependent diabetes mellitus and normal subjects Taiwan Yi Xue Hui Za Zhi 1989 88 9 883 885 2695593

[B5] Klurfeld DM Foreyt J Angelopoulos TJ Rippe JM Lack of evidence for high fructose corn syrup as the cause of the obesity epidemic Int J Obes (Lond) 2013 37 6 771 773 22986683 10.1038/ijo.2012.157 PMC3679479

[B6] Melanson KJ Zukley L Lowndes J Nguyen V Angelopoulos TJ Rippe JM Effects of high-fructose corn syrup and sucrose consumption on circulating glucose, insulin, leptin, and ghrelin and on appetite in normal-weight women Nutrition 2007 23 2 103 112 17234503 10.1016/j.nut.2006.11.001

[B7] Angelopoulos TJ Lowndes J Zukley L Melanson KJ Nguyen V Huffman A Rippe JM The effect of high-fructose corn syrup consumption on triglycerides and uric acid J Nutr 2009 139 6 1242S 1245S 19403709 10.3945/jn.108.098194

[B8] Rippe JM Kris Etherton PM Fructose, sucrose, and high fructose corn syrup: modern scientific findings and health implications Adv Nutr 2012 3 5 739 740 22983860 10.3945/an.112.002600 PMC3648763

[B9] Grynbaum M New York plans to ban sale of big sizes of sugary drinks New York Times 2012 New York May 30. pA1. [http://www.nytimes.com/2012/05/31/nyregion/bloomberg-plans-a-ban-on-large-sugared-drinks.html?pagewanted=all&_r=0]

[B10] Wescott RF Fitzpatrick BM Phillips E Industry self-regulation to improve student health: quantifying changes in beverage shipments to schools Am J Public Health 2012 102 10 1928 1935 22897528 10.2105/AJPH.2011.300610 PMC3490668

[B11] World Health Organization WHO Opens Public Consultation On Draft Sugars Guideline [ http://www.who.int/mediacentre/news/notes/2014/consultation-sugar-guideline/en/] 24844105

[B12] Han E Powell LM Consumption patterns of sugar-sweetened beverages in the United States J Acad Nutr Diet 2013 113 1 43 53 23260723 10.1016/j.jand.2012.09.016 PMC3662243

[B13] Vartanian LR Schwartz MB Brownell KD Effects of soft drink consumption on nutrition and health: a systematic review and meta-analysis Am J Public Health 2007 97 4 667 675 17329656 10.2105/AJPH.2005.083782 PMC1829363

[B14] Forshee RA Anderson PA Storey ML Sugar-sweetened beverages and body mass index in children and adolescents: a meta-analysis Am J Clin Nutr 2008 87 6 1662 1671 18541554 10.1093/ajcn/87.6.1662

[B15] Malik VS Popkin BM Bray GA Despres JP Hu FB Sugar-sweetened beverages, obesity, type 2 diabetes mellitus, and cardiovascular disease risk Circulation 2010 121 11 1356 1364 20308626 10.1161/CIRCULATIONAHA.109.876185 PMC2862465

[B16] Hu FB Malik VS Sugar-sweetened beverages and risk of obesity and type 2 diabetes: epidemiologic evidence Physiol Behav 2010 100 1 47 54 20138901 10.1016/j.physbeh.2010.01.036 PMC2862460

[B17] Gibson S Sugar-sweetened soft drinks and obesity: a systematic review of the evidence from observational studies and interventions Nutr Res Rev 2008 21 2 134 147 19087367 10.1017/S0954422408110976

[B18] Malik VS Schulze MB Hu FB Intake of sugar-sweetened beverages and weight gain: a systematic review Am J Clin Nutr 2006 84 2 274 288 16895873 10.1093/ajcn/84.1.274 PMC3210834

[B19] World Health Organization Joint WHO/FAO Expert Consultation on Diet, Nutrition and the Prevention of Chronic Diseases WHO Technical Report Series No. 916 2002 Geneva: World Health Organization 12768890

[B20] United States Department of Agriculture USDA’s Nutrition Evidence Library [ http://www.nutritionevidencelibrary.gov/]

[B21] Mattes RD Shikany JM Kaiser KA Allison DB Nutritively sweetened beverage consumption and body weight: a systematic review and meta-analysis of randomized experiments Obes Rev 2011 12 5 346 365 20524996 10.1111/j.1467-789X.2010.00755.x PMC3169649

[B22] Summerbell CD Douthwaite W Whittaker V Ells LJ Hillier F Smith S Kelly S Edmunds LD Macdonald I The association between diet and physical activity and subsequent excess weight gain and obesity assessed at 5 years of age or older: a systematic review of the epidemiological evidence Int J Obes (Lond) 2009 33 Suppl 3 S28 S34 19597430 10.1038/ijo.2009.80

[B23] Anderson GH Sugars-containing beverages and post-prandial satiety and food intake Int J Obes (Lond) 2006 30 S52 S59

[B24] Malik VS Popkin BM Bray GA Despres JP Willett WC Hu FB Sugar-sweetened beverages and risk of metabolic syndrome and type 2 diabetes: a meta-analysis Diabetes Care 2010 33 11 2477 2483 20693348 10.2337/dc10-1079 PMC2963518

[B25] Malik VS Hu FB Sugar-sweetened beverages and health: where does the evidence stand? Am J Clin Nutr 2011 94 5 1161 1162 21993436 10.3945/ajcn.111.025676 PMC3192470

[B26] Weed DL Althuis MD Mink PJ Quality of reviews on sugar-sweetened beverages and health outcomes: a systematic review Am J Clin Nutr 2011 94 5 1340 1347 21918218 10.3945/ajcn.111.015875 PMC3192479

[B27] Palmer JR Boggs DA Krishnan S Hu FB Singer M Rosenberg L Sugar-sweetened beverages and incidence of type 2 diabetes mellitus in African American women Arch Intern Med 2008 168 14 1487 1492 18663160 10.1001/archinte.168.14.1487 PMC2708080

[B28] Ruxton CH Gardner EJ McNulty HM Is sugar consumption detrimental to health? A review of the evidence 1995–2006 Crit Rev Food Sci Nutr 2010 50 1 1 19 20047137 10.1080/10408390802248569

[B29] de Ruyter JC Olthof MR Seidell JC Katan MB A trial of sugar-free or sugar-sweetened beverages and body weight in children N Engl J Med 2012 367 15 1397 1406 22998340 10.1056/NEJMoa1203034

[B30] Ebbeling CB Feldman HA Chomitz VR Antonelli TA Gortmaker SL Osganian SK Ludwig DS A randomized trial of sugar-sweetened beverages and adolescent body weight N Engl J Med 2012 367 15 1407 1416 22998339 10.1056/NEJMoa1203388 PMC3494993

[B31] Qi Q Chu AY Kang JH Jensen MK Curhan GC Pasquale LR Ridker PM Hunter DJ Willett WC Rimm EB Chasman DI Hu FB Qi L Sugar-sweetened beverages and genetic risk of obesity N Engl J Med 2012 367 15 1387 1396 22998338 10.1056/NEJMoa1203039 PMC3518794

[B32] de Ruyter JC Olthof MR Kuijper LD Katan MB Effect of sugar-sweetened beverages on body weight in children: design and baseline characteristics of the double-blind, randomized intervention study in kids Contemp Clin Trials 2012 33 1 247 257 22056980 10.1016/j.cct.2011.10.007

[B33] Hernan MA Hernandez-Diaz S Robins JM A structural approach to selection bias Epidemiology 2004 15 5 615 625 15308962 10.1097/01.ede.0000135174.63482.43

[B34] Cochrane Effective Practice and Organisation of Care Group What study designs should be included in an EPOC review and what should they be called? [ http://epoc.cochrane.org/sites/epoc.cochrane.org/files/uploads/05%20What%20study%20designs%20should%20be%20included%20in%20an%20EPOC%20review%202013%2008%2012.pdf]

[B35] Centers for Disease Control and Prevention The CDC guide to strategies for reducing the consumption of sugar-sweetened beverages [ http://www.cdph.ca.gov/SiteCollectionDocuments/StratstoReduce_Sugar_Sweetened_Bevs.pdf]

[B36] Sampson M McGowan J Cogo E Grimshaw J Moher D Lefebvre C An evidence-based practice guideline for the peer review of electronic search strategies J Clin Epidemiol 2009 62 9 944 952 19230612 10.1016/j.jclinepi.2008.10.012

[B37] Reuters T Reference Manager 12 [Computer Program] 2011 New York: Thomson Reuters

[B38] Partners E DistillerSR [Computer Program] 2011 Ottawa: Evidence Partners

[B39] Moher D Liberati A Tetzlaff J Altman DG Preferred reporting items for systematic reviews and meta-analyses: the PRISMA statement Ann Intern Med 2009 151 4 264 269 W64 19622511 10.7326/0003-4819-151-4-200908180-00135

[B40] Higgins J Altman DG Chapter 8: assessing risk of bias in included studies [ http://handbook.cochrane.org/]

[B41] Higgins J Altman DG Chapter 16: special topics in statistics [ http://handbook.cochrane.org/]

[B42] Academy of Nutrition and Dietetics Appendix 8: quality criteria checklist: primary research [ http://www.andeal.org/evidence-analysis-manual]

[B43] Cochrane Effective Practice and Organisation of Care Group Suggested risk of bias criteria for EPOC reviews [ http://epoc.cochrane.org/sites/epoc.cochrane.org/files/uploads/14%20Suggested%20risk%20of%20bias%20criteria%20for%20EPOC%20reviews%202013%2008%2012.pdf]

[B44] Balshem H Helfand M Schunemann HJ Oxman AD Kunz R Brozek J Vist GE Falck-Ytter Y Meerpohl J Norris S Guyatt GH GRADE guidelines: 3. Rating the quality of evidence J Clin Epidemiol 2011 64 4 401 406 21208779 10.1016/j.jclinepi.2010.07.015

[B45] Shrier I Platt RW Reducing bias through directed acyclic graphs BMC Med Res Methodol 2008 8 70 18973665 10.1186/1471-2288-8-70 PMC2601045

[B46] Pearl J The art and science of cause and effect Causality: Models, Reasoning and Inference 2000 Cambridge: Cambridge University 331 358

[B47] Textor J Hardt J Knuppel S DAGitty: a graphical tool for analyzing causal diagrams Epidemiology 2011 22 5 745 21811114 10.1097/EDE.0b013e318225c2be

[B48] Wiebe N Vandermeer B Platt RW Klassen TP Moher D Barrowman NJ A systematic review identifies a lack of standardization in methods for handling missing variance data J Clin Epidemiol 2006 59 4 342 353 16549255 10.1016/j.jclinepi.2005.08.017

[B49] Rosenthal R Rosnow R Rubin D Contrasts and Effect Sizes in Behavioral Research: a Correlational Approach 2000 Cambridge: Cambridge University Press

[B50] Chinn S A simple method for converting an odds ratio to effect size for use in meta-analysis Stat Med 2000 19 22 3127 3131 11113947 10.1002/1097-0258(20001130)19:22<3127::aid-sim784>3.0.co;2-m

[B51] Fu R Gartlehner G Grant M Shamliyan T Sedrakyan A Wilt TJ Griffith L Oremus M Raina P Ismaila A Santaguida P Lau J Trikalinos TA Conducting quantitative synthesis when comparing medical interventions: AHRQ and the Effective Health Care Program J Clin Epidemiol 2011 64 11 1187 1197 21477993 10.1016/j.jclinepi.2010.08.010

[B52] Borenstein M Hedges L Higgins J Rothstein H Comprehensive meta-analysis version 2 http://www.meta-analysis.com/pages/downloads/Meta-Analysis-Manual.pdf?]

[B53] SAS [ http://www.sas.com]

[B54] Ramsay CR Matowe L Grilli R Grimshaw JM Thomas RE Interrupted time series designs in health technology assessment: lessons from two systematic reviews of behavior change strategies Int J Technol Assess Health Care 2003 19 4 613 623 15095767 10.1017/s0266462303000576

[B55] DerSimonian R Laird N Meta-analysis in clinical trials Control Clin Trials 1986 7 3 177 188 3802833 10.1016/0197-2456(86)90046-2

[B56] Arends LR Hamza TH van Houwelingen JC Heijenbrok-Kal MH Hunink MG Stijnen T Bivariate random effects meta-analysis of ROC curves Med Decis Making 2008 28 5 621 638 18591542 10.1177/0272989X08319957

[B57] Deeks JJ Higgins J Altman DG Higgins JPT, Green S Analysing data and undertaking meta-analyses Cochrane Handbook for Systematic Reviews of Interventions: Cochrane Book Series 2008 Chichester: Wiley 243 296

[B58] Higgins JP Thompson SG Deeks JJ Altman DG Measuring inconsistency in meta-analyses BMJ 2003 327 7414 557 560 12958120 10.1136/bmj.327.7414.557 PMC192859

[B59] Sterne JA Sutton AJ Ioannidis JP Terrin N Jones DR Lau J Carpenter J Rucker G Harbord RM Schmid CH Tetzlaff J Deeks JJ Peters J Macaskill P Schwarzer G Duval S Altman DG Moher D Higgins JP Recommendations for examining and interpreting funnel plot asymmetry in meta-analyses of randomised controlled trials BMJ 2011 343 d4002 21784880 10.1136/bmj.d4002

[B60] Egger M Davey SG Schneider M Minder C Bias in meta-analysis detected by a simple, graphical test BMJ 1997 315 7109 629 634 9310563 10.1136/bmj.315.7109.629 PMC2127453

[B61] Rucker G Schwarzer G Carpenter J Arcsine test for publication bias in meta-analyses with binary outcomes Stat Med 2008 27 5 746 763 17592831 10.1002/sim.2971

[B62] Borenstein M Hedges LV Higgins JPT Rothstein HR Introduction to Meta-analysis 2009 New York: Wiley

[B63] Moher D Shamseer L Clarke M Reporting guidelines for systematic review protocols19th Cochrane Colloquium: 19–22 October 2011 Madrid

[B64] Shea BJ Grimshaw JM Wells GA Boers M Andersson N Hamel C Porter AC Tugwell P Moher D Bouter LM Development of AMSTAR: a measurement tool to assess the methodological quality of systematic reviews BMC Med Res Methodol 2007 7 10 17302989 10.1186/1471-2288-7-10 PMC1810543

[B65] Chow AW Epidemiology, pathogenesis and clinical manifestations of odontogenic infections [ http://www.uptodate.com/contents/epidemiology-pathogenesis-and-clinical-manifestations-of-odontogenic-infections]

[B66] Bray GA Nielsen SJ Popkin BM Consumption of high-fructose corn syrup in beverages may play a role in the epidemic of obesity Am J Clin Nutr 2004 79 4 537 543 15051594 10.1093/ajcn/79.4.537

[B67] Johnson RJ Sanchez-Lozada LG Nakagawa T The effect of fructose on renal biology and disease J Am Soc Nephrol 2010 21 12 2036 2039 21115612 10.1681/ASN.2010050506

[B68] Gagliardi AC Miname MH Santos RD Uric acid: a marker of increased cardiovascular risk Atherosclerosis 2009 202 1 11 17 18585721 10.1016/j.atherosclerosis.2008.05.022

[B69] van Dam RM Coffee consumption and risk of type 2 diabetes, cardiovascular diseases, and cancer Appl Physiol Nutr Metab 2008 33 6 1269 1283 19088789 10.1139/H08-120

[B70] Butt MS Sultan MT Coffee and its consumption: benefits and risks Crit Rev Food Sci Nutr 2011 51 4 363 373 21432699 10.1080/10408390903586412

[B71] Center for Science in the Public Interest Petition to bar the use of caramel colorings produced with ammonia and containing the carcinogens 2-methylimidazole and 4-methylimidazole [ http://cspinet.org/new/pdf/caramel_coloring_petition.pdf]

[B72] Tremblay MS Colley RC Saunders TJ Healy GN Owen N Physiological and health implications of a sedentary lifestyle Appl Physiol Nutr Metab 2010 35 6 725 740 21164543 10.1139/H10-079

[B73] Vogels TC Verrips GHW Koopman HM Theunissen NCM Fekkes M Kamphuis RP TACQOL manual. Parent form and child form 6–11 years http://worlddatabaseofhappiness.eur.nl/hap_bib/freetexts/vogels_tc_2004.pdf

