# Supplementary material for: Do sugar-sweetened beverages cause adverse health outcomes in children? A systematic review protocol
Source: Syst Rev. 2014 Sep 4;3:96. doi: 10.1186/2046-4053-3-96 (PMC4160918; doi:10.1186/2046-4053-3-96)
Supplement: Additional file 1 — Postulated physiologic mechanisms regarding disease development due to SSB consumption [65]-[72]. This file provides a description of the postulated physiologic mechanisms of disease development from the consumption of sugar-sweetened beverages. [file 2046-4053-3-96-S1.pdf]

Additional file 1

Increase in dietary glycemic load, adverse effects of endogenous compounds within SSBs such as advanced glycation end products, alterations in taste preferences, and reduced diet quality have been postulated in the development of adverse health outcomes subsequent to regular SSB consumption.[24] A high glycemic load can induce insulin resistance, β-cell dysfunction, and increase the levels of inflammatory markers such as C-reactive protein. SSBs also have a low satiety potential that may result in an overall positive energy balance.[24] Additional sugars made available in the mouth increase the risk of plaque bacteria to cause tooth decay via fermentation (reduces pH and promotes demineralization) and the development of polysaccharide polymers (promotes bacteria adherence to the tooth).[65]

In addition, most SSBs, but not all, are sweetened with high-fructose corn syrup (HFCS), which has been suggested to have particular metabolic effects that lead to obesity.[66] Fructose is a simple sugar that is absorbed and metabolized by the body by different mechanisms than glucose. Higher fructose consumption has been theorized to lead to insulin resistance, hepatic lipogenesis, and excessive activation of the glycolytic pathway.[2,4]

In addition to leading to adverse metabolic effects, fructose has been claimed to increase uric acid levels. In the liver, it has been suggested that fructose is phosphorylated through the conversion of adenosine triphosphate (ATP) to adenosine diphosphate (ADP), which is then broken down into uric acid.[67] Uric acid is associated with metabolic syndrome, hypertension, increased cardiovascular risk, gout, and chronic kidney disease.[68]

Dietary caffeine intake has been associated with reduced incidence or progression of diabetes mellitus, liver cancer, Parkinsonism, and Alzheimer’s disease, but possibly implicated in the development of dyslipidemia and cardiovascular diseases. [69,70]

Although the precise mechanisms are not known, reduced diet quality is postulated to lead to fractures and, in addition to other components of SSBs, affect neurological functioning and subsequent academic achievement.

The by-products 2-methylimidazole (2-MI) and 4-methylimidazole (4-MI) are formed during the production of caramel coloring and have been linked to cancer in studies of laboratory animals[71]; however, since the emergence of cancer in this context is unlikely to occur during the pediatric stage of life, we include it for illustrative purposes only.

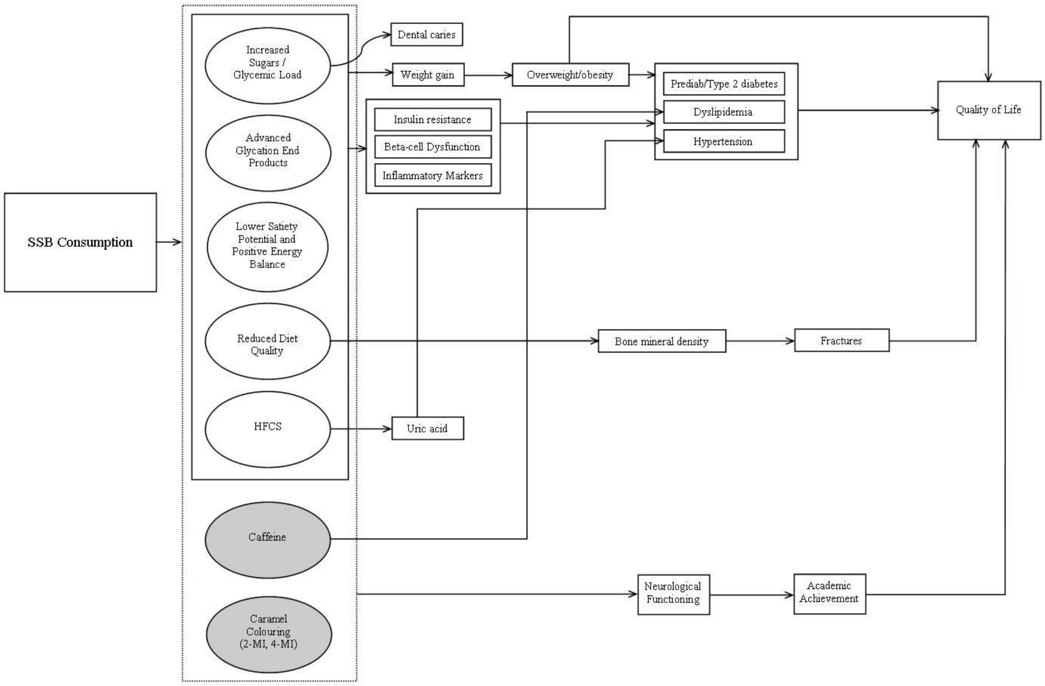

Abbreviations: HFCS = high fructose corn syrup; MI = methylimidazole; SSB = sugar-sweetened beverage. The greyed variables caffeine and 2-MI/4-MI represent beverage components present in some, but not all, SSBs.
