# Supplementary material for: Do sugar-sweetened beverages cause adverse health outcomes in children? A systematic review protocol
Source: Syst Rev. 2014 Sep 4;3:96. doi: 10.1186/2046-4053-3-96 (PMC4160918; doi:10.1186/2046-4053-3-96)
Supplement: Additional file 2 — Examples of validated generic quality of life instruments. This file provides examples of validated generic quality of life instruments. [file 2046-4053-3-96-S2.pdf]

Additional file 2

Information obtained from: PROQOLID – Patient-Reported Outcome and Quality of Life Instruments Database. http://www.proqolid.org

1. *15-Dimensional Health-Related Quality of Life Measure (15D)* - Assessment of health related quality of life and its utility in adults 16 years of age or over. A version has also been developed for adolescents 12-15 years of age (16D) and children 8-11 years of age (17D).
2. *Child Health Questionnaire (CHQ)* – Assessment of health-related quality of life in children.
3. *Euroqol EQ-5D (EQ-5D)* – Assessment of health outcomes in adults and children 12 years of age or over.
4. *Ferrans and Powers Quality of Life Index (QLI)* – Assessment of quality of life in terms of satisfaction with life.
5. *Pediatric Quality of Life Inventory Generic Core (PedsQL Generic Core Scale)* – Assessment of health-related quality of life in children who are healthy or who have acute or chronic health conditions.
6. *Pictured Child's Quality of Life Self Questionnaire (AUQUEI)* – Assessment of quality of life in children.
7. *Quality of Life Inventory (QOLI)* – Assessment of life satisfaction and outcomes based on 16 key areas of life in adolescents and adults.
8. *Quality of Life Measure for Children Aged 3-8 years (TedQL)* – Assessment of health-related quality of life in children 3-8 years of age.

9. *Short Form-12 Health Survey (SF-12)* – Shorter alternative to the SF-36 for use in adolescents (14 years of age or over) and adults.
10. *Short Form-36 Health Survey (SF-36)* – Assessment of generic health concepts in adolescents and adults.
11. *TNO-AZL Children's Quality of Life (TACQOL)* – Assessment of health-related quality of life in children.
12. *TNO-AZL Preschool Children Quality of Life Questionnaire (TAPQOL)* – Assessment of parents' perceptions of health-related quality of life in preschool children.
13. *World Health Organization Quality of Life Assessment Instrument (WHOQOL-100 and WHOQOL-BREF)* – Assessment of individuals' perceptions of quality of life.
14. *Youth Quality of Life Instrument (YQOL)* – Assessment of quality of life in youths 11-18 years of age.
