# Supplementary material for: Do sugar-sweetened beverages cause adverse health outcomes in children? A systematic review protocol
Source: Syst Rev. 2014 Sep 4;3:96. doi: 10.1186/2046-4053-3-96 (PMC4160918; doi:10.1186/2046-4053-3-96)
Supplement: Additional file 3 — Search strategy for the Embase, MEDLINE, and PsycINFO databases. This file provides the search strategies used for the various bibliographic databases. [file 2046-4053-3-96-S3.pdf]

Additional file 3

Strategies have been adapted for use in other electronic bibliographic databases.

SSB - Multifile – Trials & Etiology

2014 Mar 28

Database: Embase Classic+Embase <1947 to 2014 March 27>, Ovid MEDLINE(R) In-Process & Other Non-Indexed Citations and Ovid MEDLINE(R) <1946 to Present>, PsycINFO <1806 to March Week 4 2014> Search Strategy:

----

1 exp Carbonated Beverages/ (4020)

2 ((sugar* or sugar-sweetened or high-sugar* or sweet* or presweet* or pre-sweet* or flavor* or flavour* or non-diet or nondiet or sucrose) adj (drink* or beverage* or juice* or soda or sodas or pop or pops or refreshment* or tea or teas or chai or coffee* or punch* or milk* or water or waters)).tw. (6970)

3 coke.tw. not Coke/ (2179)

4 (cola or coca-cola or pepsi or root beer* or "Dr. Pepper" or "7up").tw. (2835)

5 (soda adj2 drink*).tw. (292)

6 (soda adj (pop or pops or beverage* or refreshment*)).tw. (159)

7 (softdrink* or soft drink* or sodapop*).tw. (5734)

8 (smoothy or smoothie*).tw. (127)

9 ((fruit* or agave or apple* or banana* or cantalope* or cherry or cherries or blackcherr* or clementine* or coconut* or cocoanut* or currant* or blackcurrant* or redcurrant* or grape* or kiwi or kiwis or kiwifruit* or lemon* or lime* or mango* or melon* or orange* or papaya* or peach* or pear* or

pineapple* or plum or plums or pomelo* or rhubarb* or tangerine* or tangelo* or watermelon* or guava* or pomegran*) adj (drink* or beverage* or juice* or nectar* or punch* or refreshment*)).tw. (16400)

10 ((berry or berries or blackberr* or blueberr* or boysonberr* or cranberr* or gooseberr* or guavaberr* or huckleberr* or loganberr* or lingonberr* or mulberr* or mullberr* or raspberr* or rumberr* or strawberr* or wineberr* or youngberr*) adj (drink* or beverage* or juice* or nectar* or punch* or refreshment*)).tw. (1199)

11 ((juice adj2 concentrat*) or juice drink* or lemonade* or limeade* or orangeade* or pineappleade* or kool aid* or koolaid*).tw. (2018)

12 (ice tea or ice teas or iced tea or iced teas or ice chai or ice chais or iced chai or iced chais or chai latte* or ice coffee* or iced coffee* or ice cappuccino* or iced cappuccino*).tw. (154)

13 Energy Drinks/ (617)

14 ((athlet* or energy or high-energy or fortif* or performance-enhancing or sport* or stamina) adj (drink* or beverage* or juice* or soda or sodas or pop or pops or refreshment* or water or waters)).tw. (2676)

15 (stimulant drink* or stimulant beverage* or gatorade* or "red bull").tw. (433)

16 (milkshake* or milk shake* or chocolate milk or strawberry milk).tw. (800)

17 (soyshake* or soy shake* or almondshake or almond shake* or dairyshake* or dairy shake*).tw. (3)

18 ((soy or almond or dairy) adj1 drink*).tw. (189)

19 (hot adj (chocolate or cocoa)).tw. (59)

20 (powder* adj drink*).tw. (66)

21 or/1-20 (39321)

22 exp Beverages/ (251205)

23 (beverage* or drink* or juice* or refreshment* or tea or teas or chai or chais or coffee* or cappuccino*).tw. (384742)

24 Fructose/ or Glucose/ or Lactose/ or Maltose/ or Sucrose/ (521822)

25 Dietary Sucrose/ (6622)

26 Sweetening Agents/ (8040)

27 (fructose or glucose or lactose or maltose or sucrose or saccharose or sugar* or sugar-sweetened or sugarcane* or agave syrup* or cane syrup* or corn syrup* or malt syrup* or honey* or molasses or sweet* or pre-sweet* or presweet* or pre-sugar* or presugar*).tw. (1134853)

28 ("d-tagatose" or tagatose).tw. (686)

29 (isomaltulose or palatinose).tw. (472)

30 Trehalose/ (7651)

31 trehalose.tw. (11253)

32 Hydrogenated starch hydrolysate*.tw. (21)

33 or/22-23 (547798)

34 or/24-32 (1286049)

35 33 and 34 (45742)

36 21 or 35 (74683)

37 (controlled clinical trial or randomized controlled trial).pt. (451118)

38 clinical trials as topic.sh. (168783)

39 (randomi#ed or randomly or RCT$1 or placebo*).tw. (1505493)

40 ((singl* or doubl* or trebl* or tripl*) adj (mask* or blind* or dumm*)).tw. (311996)

41 trial.ti. (303302)

42 or/37-41 (1914977)

43 36 and 42 (7971)

44 review.pt. (3786824)

45 43 not 44 (7700)

46 (comment or editorial or interview or letter or news).pt. (2767331)

47 45 not 46 (7676)

48 exp Animals/ not (exp Animals/ and Humans/) (8902064)

49 47 not 48 (6493)

50 time series.tw. (38426)

51 (control* adj1 ("before and after" or "before-after")).tw. (2961)

52 (pre-post or "pre test*" or pretest* or posttest* or "post test*" or (pre adj5 post)).tw. (198110)

53 (pre-intervention? or preintervention? or "pre intervention?" or postintervention? or postintervention? or "post intervention?").tw. (28318)

54 clinical trial.pt. (485371)

55 "clinical trial".tw. (191742)

56 Evaluation studies.pt. (190397)

57 (evaluation$2 adj (study or studies)).tw. (9572)

58 (control or controlled or volunteer or volunteers).tw. (5398867)

59 ((nonrandom* or non-random* or quasirandom* or quasi-random* or quasi-experiment* or quasiexperiment* or quasi-control* or quasicontrol*) adj3 (method* or study or trial or design*)).ti,ab,hw. (37304)

60 Observational study.pt. (1381)

61 (observation$2 adj (study or studies)).tw. (112882)

62 exp Cohort Studies/ (1488452)

63 cohort.tw. (627162)

64 exp Longitudinal Studies/ (936444)

65 exp Prospective Studies/ (605514)

66 exp Retrospective Studies/ (819282)

67 (longitudinal or prospective or retrospective).tw. (1802168)

68 exp Follow-Up Studies/ (1288949)

69 ((follow-up or followup) adj (study or studies)).tw. (99592)

70 ((population or population-based) adj (study or studies or analysis)).tw. (26648)

71 ((multidimensional or multi-dimensional) adj (study or studies)).tw. (287)

72 Comparative Study.pt. (1664931)

73 ((comparative or comparison) adj (study or studies)).tw. (196378)

74 exp Case-Control Studies/ (729230)

75 ((case-control* or case-based or case-comparison) adj (study or studies)).tw. (143888)

76 exp Control Groups/ (72536)

77 or/50-76 (10531064)

78 36 and 77 (23320)

79 78 not 46 (23226)

80 79 not 48 (17048)

81 49 or 80 (19010)

82 81 use prmz (8924) [MEDLINE TRIALS]

83 carbonated beverage/ (4013)

84 soft drink/ (1985)

85 ((sugar* or sugar-sweetened or high-sugar* or sweet* or presweet* or pre-sweet* or flavor* or flavour* or non-diet or nondiet or sucrose) adj (drink* or beverage* or juice* or soda or sodas or pop or pops or refreshment* or tea or teas or chai or coffee* or punch* or milk* or water or waters)).tw. (6970)

86 coke.tw. not Coke/ (2179)

87 (cola or coca-cola or pepsi or root beer* or "Dr. Pepper" or "7up").tw. (2835)

88 (soda adj2 drink*).tw. (292)

89 (soda adj (pop or pops or beverage* or refreshment*)).tw. (159)

90 (softdrink* or soft drink* or sodapop*).tw. (5734)

91 (smoothy or smoothie*).tw. (127)

92 exp fruit juice/ (7836)

93 ((fruit* or agave or apple* or banana* or cantalope* or cherry or cherries or blackcherr* or clementine* or coconut* or cocoanut* or currant* or blackcurrant* or redcurrant* or grape* or kiwi or kiwis or kiwifruit* or lemon* or lime* or mango* or melon* or orange* or papaya* or peach* or pear* or

pineapple* or plum or plums or pomelo* or rhubarb* or tangerine* or tangelo* or watermelon* or guava* or pomegran*) adj (drink* or beverage* or juice* or nectar* or punch* or refreshment*)).tw. (16400)

94 ((berry or berries or blackberr* or blueberr* or boysonberr* or cranberr* or gooseberr* or guavaberr* or huckleberr* or loganberr* or lingonberr* or mulberr* or mullberr* or raspberr* or rumberr* or strawberr* or wineberr* or youngberr*) adj (drink* or beverage* or juice* or nectar* or punch* or refreshment*)).tw. (1199)

95 ((juice adj2 concentrat*) or juice drink* or lemonade* or limeade* or orangeade* or pineappleade* or kool aid* or koolaid*).tw. (2018)

96 (ice tea or ice teas or iced tea or iced teas or ice chai or ice chais or iced chai or iced chais or chai latte* or ice coffee* or iced coffee* or ice cappuccino* or iced cappuccino*).tw. (154)

97 energy drink/ or sports drink/ (941)

98 ((athlet* or energy or high-energy or fortif* or performance-enhancing or sport* or stamina) adj (drink* or beverage* or juice* or soda or sodas or pop or pops or refreshment* or water or waters)).tw. (2676)

99 (stimulant drink* or stimulant beverage* or gatorade* or "red bull").tw. (433)

100 (milkshake* or milk shake* or chocolate milk or strawberry milk).tw. (800)

101 (soyshake* or soy shake* or almondshake or almond shake* or dairyshake* or dairy shake*).tw. (3)

102 ((soy or almond or dairy) adj1 drink*).tw. (189)

103 (hot adj (chocolate or cocoa)).tw. (59)

104 (powder* adj drink*).tw. (66)

105 or/83-104 (42168)

106 exp beverage/ (251205)

107 (beverage* or drink* or juice* or refreshment* or tea or teas or chai or chais or coffee* or cappuccino*).tw. (384742)

108 Fructose/ or Glucose/ or Lactose/ or Maltose/ or Sucrose/ (521822)

109 sugar intake/ (3990)

110 sweetening agent/ (8040)

111 (fructose or glucose or lactose or maltose or sucrose or saccharose or sugar* or sugar-sweetened or sugarcane* or agave syrup* or cane syrup* or corn syrup* or malt syrup* or honey* or molasses or sweet* or pre-sweet* or presweet* or pre-sugar* or presugar*).tw. (1134853)

112 tagatose/ (285)

113 ("d-tagatose" or tagatose).tw. (686)

114 palatinose/ (239)

115 (isomaltulose or palatinose).tw. (472)

116 Trehalose/ (7651)

117 trehalose.tw. (11253)

118 hydrogenated starch hydrolysate*.tw. (21)

119 106 or 107 (547798)

120 or/108-118 (1285657)

121 119 and 120 (45654)

122 105 or 121 (77141)

123 randomized controlled trial/ or controlled clinical trial/ (925163)

124 exp "clinical trial (topic)"/ (97336)

125 (randomi#ed or randomly or RCT$1 or placebo*).tw. (1505493)

126 ((singl* or doubl* or trebl* or tripl*) adj (mask* or blind* or dumm*)).tw. (311996)

127 trial.ti. (303302)

128 or/123-127 (2043108)

129 122 and 128 (8741)

130 review.pt. (3786824)

131 129 not 130 (8359)

132 exp animal experimentation/ or exp models animal/ or exp animal experiment/ or nonhuman/ or exp vertebrate/ (37242032)

133 exp humans/ or exp human experimentation/ or exp human experiment/ (28070152)

134 132 not 133 (9173516)

135 131 not 134 (7151)

136 (editorial or letter).pt. (2463429)

137 135 not 136 (7119)

138 time series analysis/ (13690)

139 time series.tw. (38426)

140 (control* adj1 ("before and after" or "before-after")).tw. (2961)

141 (pre-post or "pre test*" or pretest* or posttest* or "post test*" or (pre adj5 post)).tw. (198110)

142 (pre-intervention? or preintervention? or "pre intervention?" or postintervention? or postintervention? or "post intervention?").tw. (28318)

143 "clinical trial".tw. (191742)

144 exp evaluation study/ (3739)

145 (evaluation$2 adj (study or studies)).tw. (9572)

146 (control or controlled or volunteer or volunteers).tw. (5398867)

147 ((nonrandom* or non-random* or quasirandom* or quasi-random* or quasi-experiment* or quasiexperiment* or quasi-control* or quasicontrol*) adj3 (method* or study or trial or design*)).ti,ab,hw. (37304)

148 observational study/ (54366)

149 (observation$2 adj (study or studies)).tw. (112882)

150 cohort analysis/ (325484)

151 cohort.tw. (627162)

152 longitudinal study/ (149424)

153 prospective study/ (605094)

154 retrospective study/ (818942)

155 (longitudinal or prospective or retrospective).tw. (1802168)

156 follow up/ (801628)

157 ((follow-up or followup) adj (study or studies)).tw. (99592)

158 population research/ (67870)

159 ((population or population-based) adj (study or studies or analysis)).tw. (26648)

160 ((multidimensional or multi-dimensional) adj (study or studies)).tw. (287)

161 exp comparative study/ (2673233)

162 ((comparative or comparison) adj (study or studies)).tw. (196378)

163 exp case control study/ (729230)

164 ((case-control* or case-based or case-comparison) adj (study or studies)).tw. (143888)

165 control group/ (72536)

166 or/138-165 (10795284)

167 122 and 166 (24428)

168 167 not (134 or 136) (17735)

169 168 or 137 (20338)

170 169 use emczd (10808) [EMBASE TRIALS]

171 ((sugar* or sugar-sweetened or high-sugar* or sweet* or presweet* or pre-sweet* or flavor* or flavour* or non-diet or nondiet or sucrose) adj (drink* or beverage* or juice* or soda or sodas or pop or pops or refreshment* or tea or teas or chai or coffee* or punch* or milk* or water or waters)).tw. (6970)

172 coke.tw. (3233)

173 (cola or coca-cola or pepsi or root beer* or "Dr. Pepper" or "7up").tw. (2835)

174 (soda adj2 drink*).tw. (292)

175 (soda adj (pop or pops or beverage* or refreshment*)).tw. (159)

176 (softdrink* or soft drink* or sodapop*).tw. (5734)

177 (smoothy or smoothie*).tw. (127)

178 ((fruit* or agave or apple* or banana* or cantalope* or cherry or cherries or blackcherr* or clementine* or coconut* or cocoanut* or currant* or blackcurrant* or redcurrant* or grape* or kiwi or kiwis or kiwifruit* or lemon* or lime* or mango* or melon* or orange* or papaya* or peach* or pear* or pineapple* or plum or plums or pomelo* or rhubarb* or tangerine* or tangelo* or watermelon* or guava* or pomegran*) adj (drink* or beverage* or juice* or nectar* or punch* or refreshment*)).tw. (16400)

179 ((berry or berries or blackberr* or blueberr* or boysonberr* or cranberr* or gooseberr* or guavaberr* or huckleberr* or loganberr* or lingonberr* or mulberr* or mullberr* or raspberr* or rumberr* or strawberr* or wineberr* or youngberr*) adj (drink* or beverage* or juice* or nectar* or punch* or refreshment*)).tw. (1199)

180 ((juice adj2 concentrat*) or juice drink* or lemonade* or limeade* or orangeade* or pineappleade* or kool aid* or koolaid*).tw. (2018)

181 (ice tea or ice teas or iced tea or iced teas or ice chai or ice chais or iced chai or iced chais or chai latte* or ice coffee* or iced coffee* or ice cappuccino* or iced cappuccino*).tw. (154)

182 ((athlet* or energy or high-energy or fortif* or performance-enhancing or sport* or stamina) adj (drink* or beverage* or juice* or soda or sodas or pop or pops or refreshment* or water or waters)).tw. (2676)

183 (stimulant drink* or stimulant beverage* or gatorade* or "red bull").tw. (433)

184 (milkshake* or milk shake* or chocolate milk or strawberry milk).tw. (800)

185 (soyshake* or soy shake* or almondshake or almond shake* or dairyshake* or dairy shake*).tw. (3)

186 ((soy or almond or dairy) adj1 drink*).tw. (189)

187 (hot adj (chocolate or cocoa)).tw. (59)

188 (powder* adj drink*).tw. (66)

189 or/171-188 (38704)

190 "beverages (nonalcoholic)"/ (907)

191 (beverage* or drink* or juice* or refreshment* or tea or teas or chai or chais or coffee* or cappuccino*).tw. (384742)

192 Sugars/ (88936)

193 glucose/ (426839)

194 (fructose or glucose or lactose or maltose or sucrose or saccharose or sugar* or sugar-sweetened or sugarcane* or agave syrup* or cane syrup* or corn syrup* or malt syrup* or honey* or molasses or sweet* or pre-sweet* or presweet* or pre-sugar* or presugar*).tw. (1134853)

195 ("d-tagatose" or tagatose).tw. (686)

196 (isomaltulose or palatinose).tw. (472)

197 trehalose.tw. (11253)

198 hydrogenated starch hydrolysate.tw. (17)

199 190 or 191 (384781)

200 or/192-198 (1307123)

201 199 and 200 (32496)

202 189 or 201 (61266)

203 clinical trials/ (46219)

204 (randomi#ed or randomly or RCT$1 or placebo*).tw. (1505493)

205 ((singl* or doubl* or trebl* or tripl*) adj (mask* or blind* or dumm*)).tw. (311996)

206 trial.ti. (303302)

207 or/203-206 (1692275)

208 202 and 207 (6299)

209 exp Animals/ not (exp Animals/ and Humans/) (8902064)

210 208 not 209 (5661)

211 time series/ (13511)

212 time series.tw. (38426)

213 (control* adj1 ("before and after" or "before-after")).tw. (2961)

214 (pre-post or "pre test*" or pretest* or posttest* or "post test*" or (pre adj5 post)).tw. (198110)

215 (pre-intervention? or preintervention? or "pre intervention?" or postintervention? or postintervention? or "post intervention?").tw. (28318)

216 "clinical trial".tw. (191742)

217 (evaluation$2 adj (study or studies)).tw. (9572)

218 (control or controlled or volunteer or volunteers).tw. (5398867)

219 ((nonrandom* or non-random* or quasirandom* or quasi-random* or quasi-experiment* or quasiexperiment* or quasi-control* or quasicontrol*) adj3 (method* or study or trial or design*)).ti,ab,hw. (37304)

220 (observation$2 adj (study or studies)).tw. (112882)

221 cohort analysis/ (325484)

222 cohort.tw. (627162)

223 exp longitudinal studies/ (936444)

224 retrospective studies/ (819282)

225 (longitudinal or prospective or retrospective).tw. (1802168)

226 followup studies/ (499628)

227 ((follow-up or followup) adj (study or studies)).tw. (99592)

228 ((population or population-based) adj (study or studies or analysis)).tw. (26648)

229 ((multidimensional or multi-dimensional) adj (study or studies)).tw. (287)

230 ((comparative or comparison) adj (study or studies)).tw. (196378)

231 ((case-control* or case-based or case-comparison) adj (study or studies)).tw. (143888)

232 experiment controls/ (695)

233 or/211-232 (8575291)

234 202 and 233 (17364)

235 234 not 209 (13164)

236 210 or 235 (15471)

237 236 use prmz (6345)

238 236 use emczd (8265)

239 236 not (237 or 238) (861) [PSYCINFO TRIALS]

240 82 or 170 or 239 (20593) [TRIALS – ALL DATABASES]

241 limit 240 to yr="2012-current" (4295)

242 limit 240 to yr="2008-2011" (5839)

243 limit 240 to yr="2001-2007" (5161)

244 limit 240 to yr="1990-2000" (3337)

245 240 not (241 or 242 or 243 or 244) (1961)

246 remove duplicates from 241 (3126)

247 remove duplicates from 242 (3969)

248 remove duplicates from 243 (3240)

249 remove duplicates from 244 (2092)

250 remove duplicates from 245 (1467)

251 246 or 247 or 248 or 249 or 250 (13894) [UNIQUE TRIALS – ALL DATABASES]

252 251 use prmz (8768) [UNIQUE TRIALS - MEDLINE]

253 251 use emczd (4798) [UNIQUE TRIALS - EMBASE]

254 251 not (252 or 253) (328) [UNIQUE TRIALS - PSYCINFO]

255 (risk or mortality).mp. or cohort.tw. use prmz (4967341) [MEDLINE ETIOLOGY FILTER]

256 36 and 255 (10410)

257 256 not (46 or 48) (9251)

258 257 use prmz (3666) [MEDLINE ETIOLOGY]

259 (risk or mortalit* or cohort).tw. use emczd (2229073) [EMBASE ETIOLOGY FILTER]

260 122 and 259 (5790)

261 260 not (134 or 136) (5176) [EMBASE ETIOLOGY]

262 (risk or mortalit* or cohort).tw. (4137185)

263 202 and 262 (8757)

264 263 not 209 (8019)

265 264 use prmz (3232)

266 264 use emczd (4430)

267 264 not (265 or 266) (357) [PSYCINFO ETIOLOGY]

268 261 use emczd (5176) [EMBASE ETIOLOGY – CONFIRMED AS PER LINE 261]

269 258 or 267 or 268 (9199) [ETIOLOGY – ALL DATABASES]

270 269 not 240 (4073) [OVERLAP WITH TRIAL SET, REMOVED]

271 remove duplicates from 270 (2945) [UNIQUE ETIOLOGY – ALL DATABASES]

272 251 or 271 (16839) [TRIALS & ETIOLOGY – ALL DATABASES]

273 272 use prmz (10305) [MEDLINE UNIQUE RECORDS]

274 272 use emczd (6125) [EMBASE UNIQUE RECORDS]

275 272 not (273 or 274) (409) [PSYCINFO UNIQUE RECORDS]

****

SSBs – Reviews

2014 Mar 26

Database: Embase Classic+Embase <1947 to 2014 March 25>, Ovid MEDLINE(R) In-Process & Other Non-Indexed Citations and Ovid MEDLINE(R) <1946 to Present>, PsycINFO <1806 to March Week 3 2014> Search Strategy:

----

1 exp Carbonated Beverages/ (4018)

2 ((sugar* or sugar-sweetened or high-sugar* or sweet* or presweet* or pre-sweet* or flavor* or flavour* or non-diet or nondiet or sucrose) adj (drink* or beverage* or juice* or soda or sodas or pop or pops or refreshment* or tea or teas or chai or coffee* or punch* or milk* or water or waters)).tw. (6964)

3 coke.tw. not Coke/ (2180)

4 (cola or coca-cola or pepsi or root beer* or "Dr. Pepper" or "7up").tw. (2834)

5 (soda adj2 drink*).tw. (292)

6 (soda adj (pop or pops or beverage* or refreshment*)).tw. (159)

7 (softdrink* or soft drink* or sodapop*).tw. (5728)

8 (smoothy or smoothie*).tw. (127)

9 ((fruit* or agave or apple* or banana* or cantalope* or cherry or cherries or blackcherr* or clementine* or coconut* or cocoanut* or currant* or blackcurrant* or redcurrant* or grape* or kiwi or kiwis or kiwifruit* or lemon* or lime* or mango* or melon* or orange* or papaya* or peach* or pear* or pineapple* or plum or plums or pomelo* or rhubarb* or tangerine* or tangelo* or watermelon* or guava* or pomegran*) adj (drink* or beverage* or juice* or nectar* or punch* or refreshment*)).tw. (16388)

10 ((berry or berries or blackberr* or blueberr* or boysonberr* or cranberr* or gooseberr* or guavaberr* or huckleberr* or loganberr* or lingonberr* or mulberr* or mullberr* or raspberr* or rumberr* or strawberr* or wineberr* or youngberr*) adj (drink* or beverage* or juice* or nectar* or punch* or refreshment*)).tw. (1198)

11 ((juice adj2 concentrat*) or juice drink* or lemonade* or limeade* or orangeade* or pineappleade* or kool aid* or koolaid*).tw. (2019)

12 (ice tea or ice teas or iced tea or iced teas or ice chai or ice chais or iced chai or iced chais or chai latte* or ice coffee* or iced coffee* or ice cappuccino* or iced cappuccino*).tw. (153)

13 Energy Drinks/ (617)

14 ((athlet* or energy or high-energy or fortif* or performance-enhancing or sport* or stamina) adj (drink* or beverage* or juice* or soda or sodas or pop or pops or refreshment* or water or waters)).tw. (2674)

15 (stimulant drink* or stimulant beverage* or gatorade* or "red bull").tw. (433)

16 (milkshake* or milk shake* or chocolate milk or strawberry milk).tw. (800)

17 (soyshake* or soy shake* or almondshake or almond shake* or dairyshake* or dairy shake*).tw. (3)

18 ((soy or almond or dairy) adj1 drink*).tw. (189)

19 (hot adj (chocolate or cocoa)).tw. (59)

20 (powder* adj drink*).tw. (66)

21 or/1-20 (39294)

22 exp Beverages/ (250992)

23 (beverage* or drink* or juice* or refreshment* or tea or teas or chai or chais or coffee* or cappuccino*).tw. (384485)

24 Fructose/ or Glucose/ or Lactose/ or Maltose/ or Sucrose/ (521422)

25 Dietary Sucrose/ (6616)

26 Sweetening Agents/ (8034)

27 (fructose or glucose or lactose or maltose or sucrose or saccharose or sugar* or sugar-sweetened or sugarcane* or agave syrup* or cane syrup* or corn syrup* or malt syrup* or honey* or molasses or sweet* or pre-sweet* or presweet* or pre-sugar* or presugar*).tw. (1134151)

28 ("d-tagatose" or tagatose).tw. (686)

29 (isomaltulose or palatinose).tw. (472)

30 Trehalose/ (7647)

31 trehalose.tw. (11253)

32 Hydrogenated starch hydrolysate*.tw. (21)

33 or/22-23 (547435)

34 or/24-32 (1285264)

35 33 and 34 (45712)

36 21 or 35 (74632)

37 limit 36 to systematic reviews [Limit not valid in Embase; records were retained] (41193)

38 meta analysis.pt. (45862)

39 meta-analysis/ (126072)

40 exp meta-analysis as topic/ (25813)

41 (meta-analy* or metanaly* or metaanaly* or met analy* or integrative research or integrative review* or integrative overview* or research integration or research overview* or collaborative review*).tw. (162180)

42 (systematic review* or systematic overview* or evidence-based review* or evidence-based overview* or (evidence adj3 (review* or overview*)) or meta-review* or meta-overview* or meta-synthes* or "review of reviews" or technology assessment* or HTA or HTAs).tw. (196262)

43 exp Technology assessment, biomedical/ (20451)

44 (cochrane or health technology assessment or evidence report).jw. (24152)

45 or/38-44 (393406)

46 36 and 45 (651)

47 37 or 46 (41286)

48 exp Animals/ not (exp Animals/ and Humans/) (8897765)

49 47 not 48 (31507)

50 (comment or editorial or interview or letter or news).pt. (2766199)

51 49 not 50 (30834)

52 51 use prmz (362) [MEDLINE REVIEWS]

53 carbonated beverage/ (4011)

54 soft drink/ (1979)

55 ((sugar* or sugar-sweetened or high-sugar* or sweet* or presweet* or pre-sweet* or flavor* or flavour* or non-diet or nondiet or sucrose) adj (drink* or beverage* or juice* or soda or sodas or pop or pops or refreshment* or tea or teas or chai or coffee* or punch* or milk* or water or waters)).tw. (6964)

56 coke.tw. not Coke/ (2180)

57 (cola or coca-cola or pepsi or root beer* or "Dr. Pepper" or "7up").tw. (2834)

58 (soda adj2 drink*).tw. (292)

59 (soda adj (pop or pops or beverage* or refreshment*)).tw. (159)

60 (softdrink* or soft drink* or sodapop*).tw. (5728)

61 (smoothy or smoothie*).tw. (127)

62 exp fruit juice/ (7833)

63 ((fruit* or agave or apple* or banana* or cantalope* or cherry or cherries or blackcherr* or clementine* or coconut* or cocoanut* or currant* or blackcurrant* or redcurrant* or grape* or kiwi or kiwis or kiwifruit* or lemon* or lime* or mango* or melon* or orange* or papaya* or peach* or pear* or pineapple* or plum or plums or pomelo* or rhubarb* or tangerine* or tangelo* or watermelon* or guava* or pomegran*) adj (drink* or beverage* or juice* or nectar* or punch* or refreshment*)).tw. (16388)

64 ((berry or berries or blackberr* or blueberr* or boysonberr* or cranberr* or gooseberr* or guavaberr* or huckleberr* or loganberr* or lingonberr* or mulberr* or mullberr* or raspberr* or rumberr* or strawberr* or wineberr* or youngberr*) adj (drink* or beverage* or juice* or nectar* or punch* or refreshment*)).tw. (1198)

65 ((juice adj2 concentrat*) or juice drink* or lemonade* or limeade* or orangeade* or pineappleade* or kool aid* or koolaid*).tw. (2019)

66 (ice tea or ice teas or iced tea or iced teas or ice chai or ice chais or iced chai or iced chais or chai latte* or ice coffee* or iced coffee* or ice cappuccino* or iced cappuccino*).tw. (153)

67 energy drink/ or sports drink/ (941)

68 ((athlet* or energy or high-energy or fortif* or performance-enhancing or sport* or stamina) adj (drink* or beverage* or juice* or soda or sodas or pop or pops or refreshment* or water or waters)).tw. (2674)

69 (stimulant drink* or stimulant beverage* or gatorade* or "red bull").tw. (433)

70 (milkshake* or milk shake* or chocolate milk or strawberry milk).tw. (800)

71 (soyshake* or soy shake* or almondshake or almond shake* or dairyshake* or dairy shake*).tw. (3)

72 ((soy or almond or dairy) adj1 drink*).tw. (189)

73 (hot adj (chocolate or cocoa)).tw. (59)

74 (powder* adj drink*).tw. (66)

75 or/53-74 (42138)

76 exp beverage/ (250992)

77 (beverage* or drink* or juice* or refreshment* or tea or teas or chai or chais or coffee* or cappuccino*).tw. (384485)

78 Fructose/ or Glucose/ or Lactose/ or Maltose/ or Sucrose/ (521422)

79 sugar intake/ (3988)

80 sweetening agent/ (8034)

81 (fructose or glucose or lactose or maltose or sucrose or saccharose or sugar* or sugar-sweetened or sugarcane* or agave syrup* or cane syrup* or corn syrup* or malt syrup* or honey* or molasses or sweet* or pre-sweet* or presweet* or pre-sugar* or presugar*).tw. (1134151)

82 tagatose/ (285)

83 ("d-tagatose" or tagatose).tw. (686)

84 palatinose/ (239)

85 (isomaltulose or palatinose).tw. (472)

86 Trehalose/ (7647)

87 trehalose.tw. (11253)

88 hydrogenated starch hydrolysate*.tw. (21)

89 76 or 77 (547435)

90 or/78-88 (1284874)

91 89 and 90 (45625)

92 75 or 91 (77089)

93 meta-analysis/ (126072)

94 "systematic review"/ (72310)

95 "meta analysis (topic)"/ (12282)

96 (meta-analy* or metanaly* or metaanaly* or met analy* or integrative research or integrative review* or integrative overview* or research integration or research overview* or collaborative review*).tw. (162180)

97 (systematic review* or systematic overview* or evidence-based review* or evidence-based overview* or (evidence adj3 (review* or overview*)) or meta-review* or meta-overview* or meta-synthes* or "review of reviews" or technology assessment* or HTA or HTAs).tw. (196262)

98 biomedical technology assessment/ (19353)

99 (cochrane or health technology assessment or evidence report).jw. (24152)

100 or/93-99 (400825)

101 92 and 100 (760)

102 exp animals/ or exp animal experimentation/ or exp models animal/ or exp animal experiment/ or nonhuman/ or exp vertebrate/ (38012541)

103 exp humans/ or exp human experimentation/ or exp human experiment/ (28047847)

104 102 not 103 (9966304)

105 101 not 104 (741)

106 (editorial or letter).pt. (2462398)

107 105 not 106 (726)

108 107 use emczd (464) [EMBASE REVIEWS]

109 ((sugar* or sugar-sweetened or high-sugar* or sweet* or presweet* or pre-sweet* or flavor* or flavour* or non-diet or nondiet or sucrose) adj (drink* or beverage* or juice* or soda or sodas or pop or pops or refreshment* or tea or teas or chai or coffee* or punch* or milk* or water or waters)).tw. (6964)

110 coke.tw. (3231)

111 (cola or coca-cola or pepsi or root beer* or "Dr. Pepper" or "7up").tw. (2834)

112 (soda adj2 drink*).tw. (292)

113 (soda adj (pop or pops or beverage* or refreshment*)).tw. (159)

114 (softdrink* or soft drink* or sodapop*).tw. (5728)

115 (smoothy or smoothie*).tw. (127)

116 ((fruit* or agave or apple* or banana* or cantalope* or cherry or cherries or blackcherr* or clementine* or coconut* or cocoanut* or currant* or blackcurrant* or redcurrant* or grape* or kiwi or

kiwis or kiwifruit* or lemon* or lime* or mango* or melon* or orange* or papaya* or peach* or pear* or pineapple* or plum or plums or pomelo* or rhubarb* or tangerine* or tangelo* or watermelon* or guava* or pomegran*) adj (drink* or beverage* or juice* or nectar* or punch* or refreshment*)).tw. (16388)

117 ((berry or berries or blackberr* or blueberr* or boysonberr* or cranberr* or gooseberr* or guavaberr* or huckleberr* or loganberr* or lingonberr* or mulberr* or mullberr* or raspberr* or rumberr* or strawberr* or wineberr* or youngberr*) adj (drink* or beverage* or juice* or nectar* or punch* or refreshment*)).tw. (1198)

118 ((juice adj2 concentrat*) or juice drink* or lemonade* or limeade* or orangeade* or pineappleade* or kool aid* or koolaid*).tw. (2019)

119 (ice tea or ice teas or iced tea or iced teas or ice chai or ice chais or iced chai or iced chais or chai latte* or ice coffee* or iced coffee* or ice cappuccino* or iced cappuccino*).tw. (153)

120 ((athlet* or energy or high-energy or fortif* or performance-enhancing or sport* or stamina) adj (drink* or beverage* or juice* or soda or sodas or pop or pops or refreshment* or water or waters)).tw. (2674)

121 (stimulant drink* or stimulant beverage* or gatorade* or "red bull").tw. (433)

122 (milkshake* or milk shake* or chocolate milk or strawberry milk).tw. (800)

123 (soyshake* or soy shake* or almondshake or almond shake* or dairyshake* or dairy shake*).tw. (3)

124 ((soy or almond or dairy) adj1 drink*).tw. (189)

125 (hot adj (chocolate or cocoa)).tw. (59)

126 (powder* adj drink*).tw. (66)

127 or/109-126 (38674)

128 "beverages (nonalcoholic)"/ (903)

129 (beverage* or drink* or juice* or refreshment* or tea or teas or chai or chais or coffee* or cappuccino*).tw. (384485)

130 Sugars/ (88892)

131 glucose/ (426499)

132 (fructose or glucose or lactose or maltose or sucrose or saccharose or sugar* or sugar-sweetened or sugarcane* or agave syrup* or cane syrup* or corn syrup* or malt syrup* or honey* or molasses or sweet* or pre-sweet* or presweet* or pre-sugar* or presugar*).tw. (1134151)

133 ("d-tagatose" or tagatose).tw. (686)

134 (isomaltulose or palatinose).tw. (472)

135 trehalose.tw. (11253)

136 hydrogenated starch hydrolysate.tw. (17)

137 128 or 129 (384524)

138 or/130-136 (1306327)

139 137 and 138 (32477)

140 127 or 139 (61221)

141 limit 140 to "0830 systematic review" [Limit not valid in Embase,Ovid MEDLINE(R),Ovid MEDLINE(R) In-Process; records were retained] (57332)

142 meta analysis/ (126072)

143 (meta-analy* or metanaly* or metaanaly* or met analy* or integrative research or integrative review* or integrative overview* or research integration or research overview* or collaborative review*).tw. (162180)

144 (systematic review* or systematic overview* or evidence-based review* or evidence-based overview* or (evidence adj3 (review* or overview*)) or meta-review* or meta-overview* or meta-synthes* or "review of reviews" or technology assessment* or HTA or HTAs).tw. (196262)

145 or/142-144 (354222)

146 140 and 145 (515)

147 141 or 146 (57346)

148 exp Animals/ not (exp Animals/ and Humans/) (8897765)

149 147 not 148 (45883)

150 149 use prmz (19372)

151 149 use emczd (26489)

152 149 not (150 or 151) (22) [PSYCINFO REVIEWS]

153 52 or 108 or 152 (848) [TOTAL HITS BEFORE DE-DUPING]

154 remove duplicates from 153 (633) [TOTAL UNIQUE HITS]

155 154 use prmz (339) [MEDLINE UNIQUE HITS]

156 154 use emczd (289) [EMBASE UNIQUE HITS]

157 154 not (155 or 156) (5) [PSYCINFO UNIQUE HITS]
