# Supplementary material for: Do sugar-sweetened beverages cause adverse health outcomes in children? A systematic review protocol
Source: Syst Rev. 2014 Sep 4;3:96. doi: 10.1186/2046-4053-3-96 (PMC4160918; doi:10.1186/2046-4053-3-96)
Supplement: Additional file 5 — Causal directed acyclic graphs (DAG) depicting the postulated causal and biasing pathways between sugar-sweetened beverage consumption and adverse health outcomes [72],[73]. This file provides illustrations and accompanying text of the postulated causal and biasing pathways between sugar-sweetened beverage consumption and adverse health outcomes. [file 2046-4053-3-96-S5.pdf]

Additional file 5

Causal DAG for selected outcomes in children

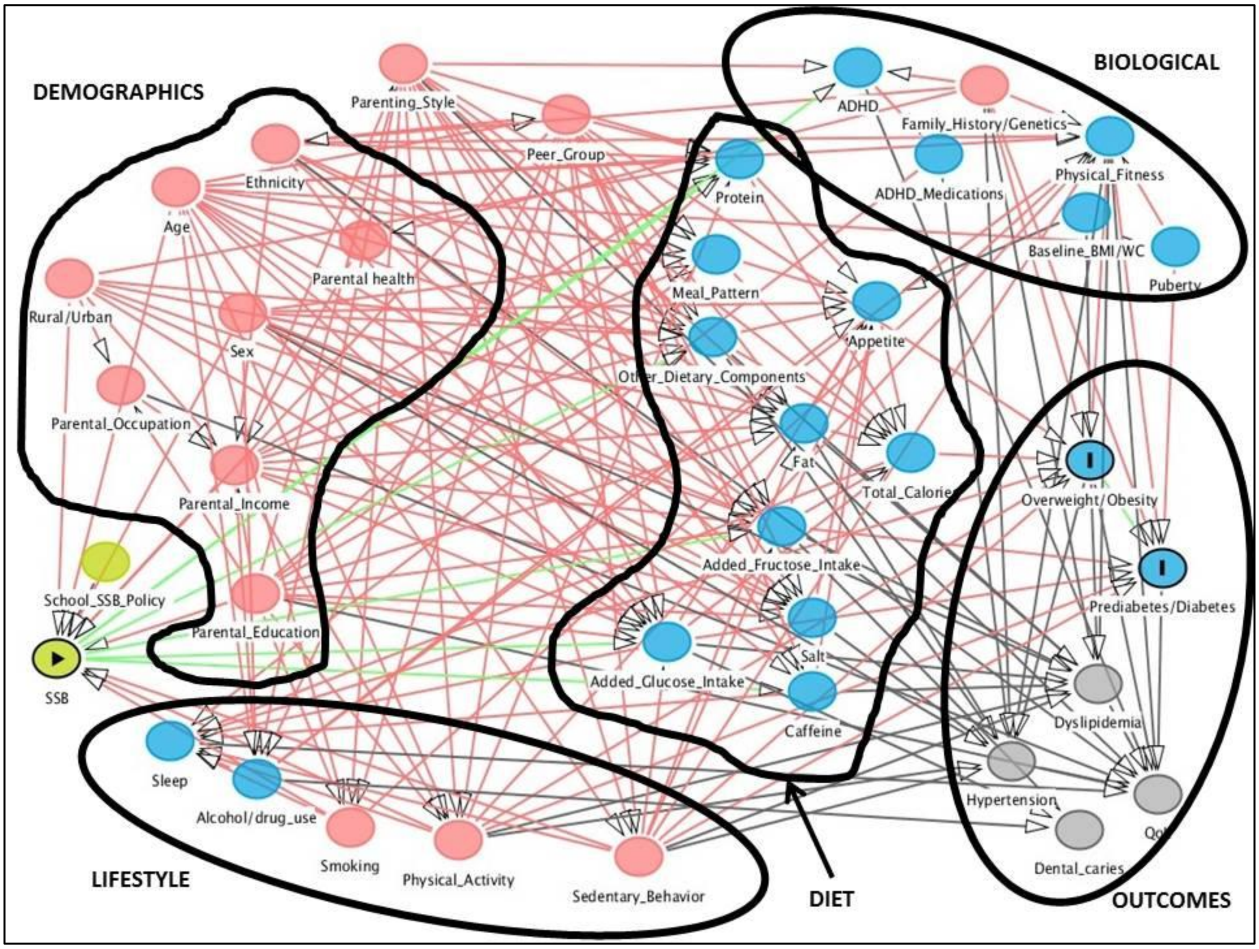

Legend: Exposure Outcome Ancestor of exposure Ancestor of outcome

Ancestor of exposure *and* outcome

Irrelevant variable. Intrinsic to the Dagitty program irrelevant variables appear because they are implicated in a causal or biasing pathway for another outcome in the DAG figure.

Causal path Biasing path

Abbreviations: ADHD = attention deficit hyperactivity disorder; BMI = body mass index; QoL = quality of life; SSB = sugar-sweetened beverage; WC = waist circumference

Minimal sufficient adjustment set for estimating the total effect of SSB consumption on each of the indicated outcomes: Age, Parental Education, Parental Income, Peer Group, Physical Activity, Rural/Urban, Sedentary Behavior, Sex, Smoking

This figure displays the DAG for metabolic-related diseases and quality of life in the pediatric population.

Within the demographic variables, parental income, parental education, and parental occupation together describe socioeconomic status. Rural/urban has been included to take into account the effect of the physical environment on disease. Upon reflection, it came to light that there may be a finer distinction to the physical environment than rural/urban, denoted by the neighborhood environment. The neighborhood environment may include factors such as proximity to or density of fast food restaurants and physical recreation facilities. We have currently not included neighborhood environment in the DAG but we will consider doing so on a post-hoc basis if we find that such variables are considered in studies. Since neighborhood environment will likely be assessed at the aggregate level, the issue of ecological fallacy will need to be taken into consideration if it is included post-hoc.

Peer group refers to the behaviours or disease status (e.g. overweight/obese) of subjects' friends.

Meal pattern refers to time of day at which and number of meals are consumed.

Within the lifestyle variables, activity level has been divided into physical activity and sedentary behavior. Sedentary behavior is defined as a distinct class of behaviours (for example, watching television or driving) with little physical movement and low energy expenditure ($\leq$ 1.5 metabolic equivalents).[72] Studies have shown a dose-response relationship between sedentary activities and adverse health outcomes.[72] Physical fitness, being a functional measure, is also considered in these pathways but as a biological variable.

The direct arrow from SSB consumption to ADHD is meant to portray the possibility that SSB may worsen the progression of pre-existing ADHD rather than causing new-incidence ADHD. The effect of

the indicated diseases on quality of life will likely be mediated through other variables not shown in the DAG. The assessment of quality of life in the pediatric population may include some child-specific factors, such as play, peer acceptance, and self-care.[73] In addition to the direct links between disease and quality of life, we have depicted direct links from parenting style, ethnicity, parental education, and parental occupation to quality of life. Again, these factors will likely act through other domains, such as peer acceptance.

Causal DAG for fracture in children

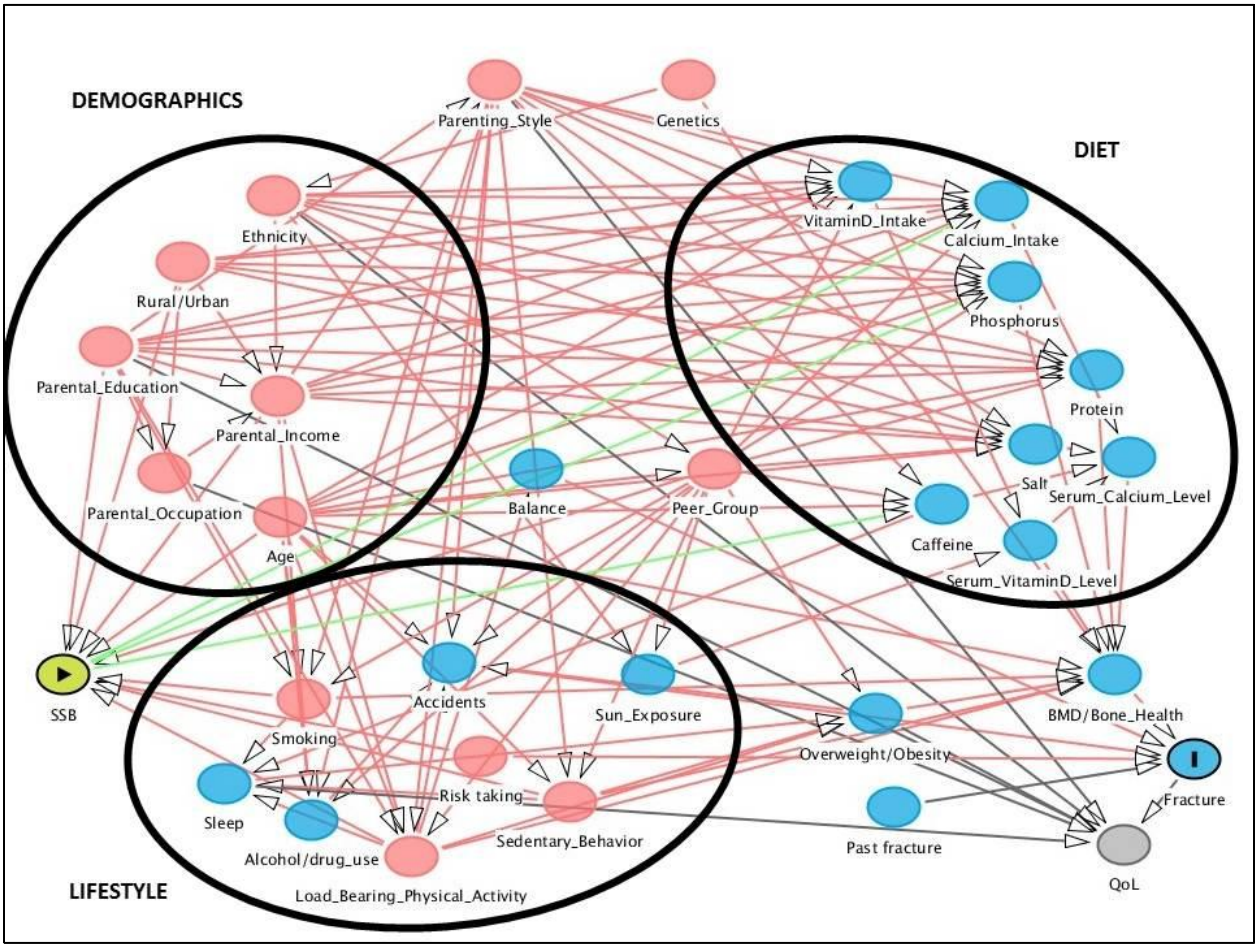

Abbreviations: BMD = bone mineral density; QoL = quality of life; SSB = sugar-sweetened beverage

Minimal sufficient adjustment set for estimating the total effect of SSB consumption on fracture and quality of life: Age, Load Bearing Physical Activity, Parental Education, Parental Income, Peer Group, Risk taking, Rural/Urban, Sedentary Behavior, Smoking

Figure 1 displays the DAG for fracture and quality of life in the pediatric population. Under the dietary variables, we have included only the most important vitamins and minerals that affect bone health (i.e. calcium, phosphorus, and vitamin D). We recognize that several other dietary components play a role in maintaining bone health (e.g. vitamin K, boron, magnesium, potassium). These have not been included in the DAG because they will likely not add much in utility to studies that have adjusted for the primary vitamins and minerals.

When quality of life is assessed in the context of the incidence of fractures, the minimum adjustment set shown here will be used for the assessment of study confounding bias.

Causal DAG for academic achievement in children

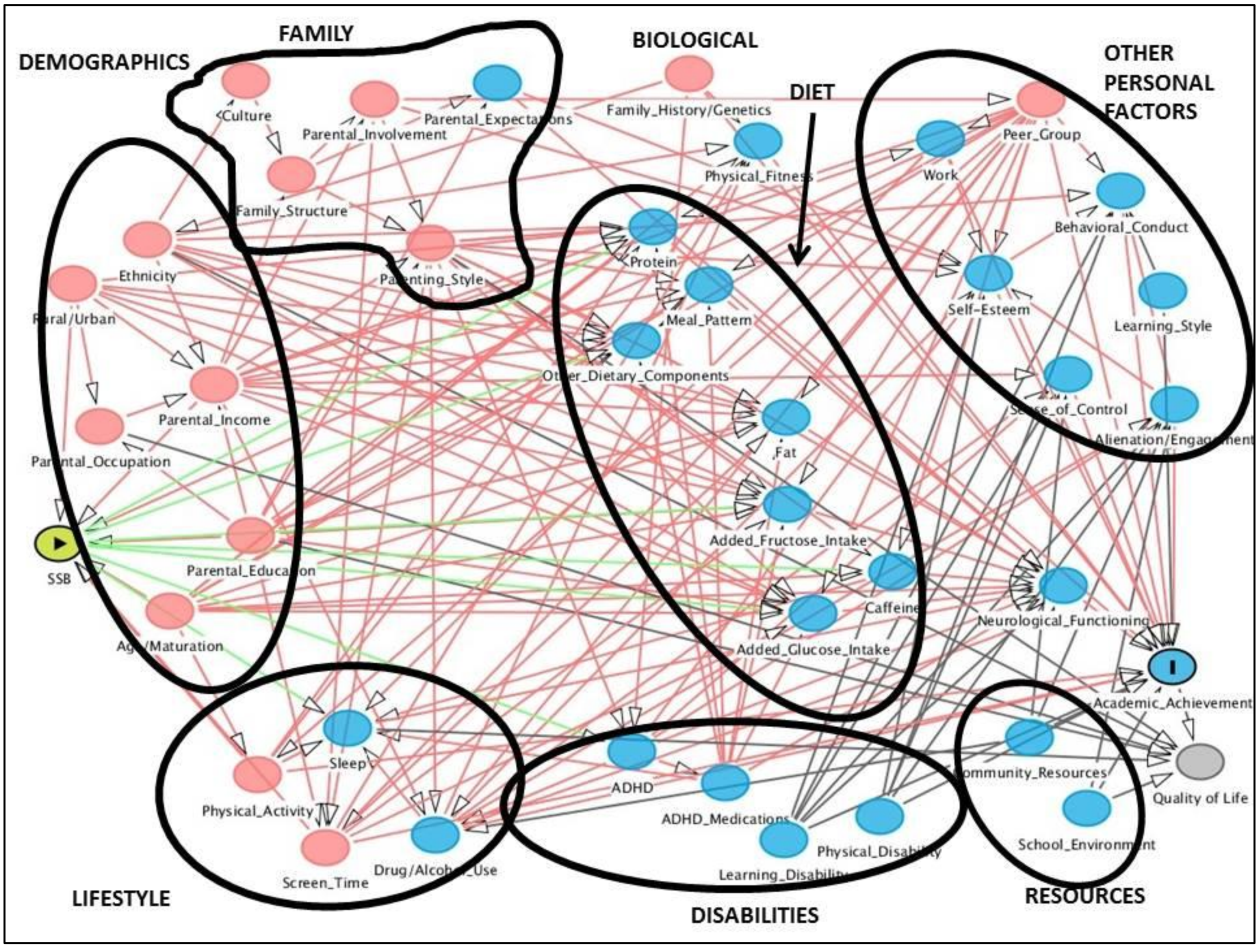

Abbreviations: ADHD = attention deficit hyperactivity disorder; SSB = sugar-sweetened beverage

Minimal sufficient adjustment set for estimating the total effect of SSB consumption on academic achievement: Age/Maturation, Parental Education, Parental Income, Peer Group, Physical Activity, Rural/Urban, Screen Time

The figure above displays the DAG for academic achievement in children. As with Box 1, the link from SSB to ADHD is meant to indicate progression of ADHD rather than new-onset ADHD. The variable

school environment may refer to factors such as class size, teacher-student ratio, extra-curricular programs, and availability of resources for teachers or students.

When quality of life is assessed in the context of the incidence of fractures, the minimum adjustment set shown here will be used for the assessment of study confounding bias.
